# Supplementary material for: Local polar order controls mechanical stress and triggers layer formation in Myxococcus xanthus colonies
Source: Nat Commun. 2025 Jan 22;16:952. doi: 10.1038/s41467-024-55806-6 (PMC11754464; doi:10.1038/s41467-024-55806-6)
Supplement: Supplementary file 1 — Supplementary Information [file 41467_2024_55806_MOESM1_ESM.pdf]

Supplemental Information

Local polar order controls mechanical stress and triggers layer formation in *Myxococcus xanthus* colonies

Endao Han,[1,2,*] Chenyi Fei,[3,4] Ricard Alert,[5,6,7] Katherine Copenhagen,[3]
Matthias D. Koch,[3,4] Ned S. Wingreen,[3,4] and Joshua W. Shaevitz[1,3,†]

[1] *Joseph Henry Laboratories of Physics, Princeton University, Princeton, NJ 08544, USA.*
[2] *School of Physical and Mathematical Sciences, Nanyang Technological University, 637371, Singapore*
[3] *Lewis-Sigler Institute for Integrative Genomics, Princeton University, Princeton, NJ 08544, USA.*
[4] *Department of Molecular Biology, Princeton University, Princeton, NJ 08544, USA.*
[5] *Max Planck Institute for the Physics of Complex Systems, Nöthnitzerstraße 38, 01187 Dresden, Germany.*
[6] *Center for Systems Biology Dresden, Pfotenhauerstraße 108, 01307 Dresden, Germany.*
[7] *Cluster of Excellence Physics of Life, TU Dresden, 01062, Dresden, Germany.*

Contents

I. Extended data 2

II. Theories on traction and cell flow near defects 9
- A. Landau-de Gennes theory 9
- B. Hydrodynamics of two-dimensional incompressible cell monolayers 9
- C. Velocity and traction around topological defects 12

III. Bacterial strains 14

IV. Measuring director and velocity fields from images of cells 17
- A. Processing bright field images of the cells 17
- B. Detecting holes in cell layers 17
- C. Nematic order 18
- D. Detection and tracking of topological defects 18
- E. Measuring velocity and cell flows 19

V. Measuring cell polarity 20
- A. Single-cell polarity vs. velocity 20
- B. Data processing for cell layers 20

VI. Measuring traction from fluorescence images 21
- A. Pre-processing fluorescence images 22
- B. Measuring displacement at the substrate surface 23
- C. Traction reconstruction 23
- D. Optimizing the regularization parameter 25

VII. Deformation normal to the substrate surface 26
- A. Measuring normal deformation of the substrate surface 26
- B. Zero traction using layer information 27

VIII. Supplementary Tables 29

IX. Supplemental Movies 30

References 31

I. EXTENDED DATA

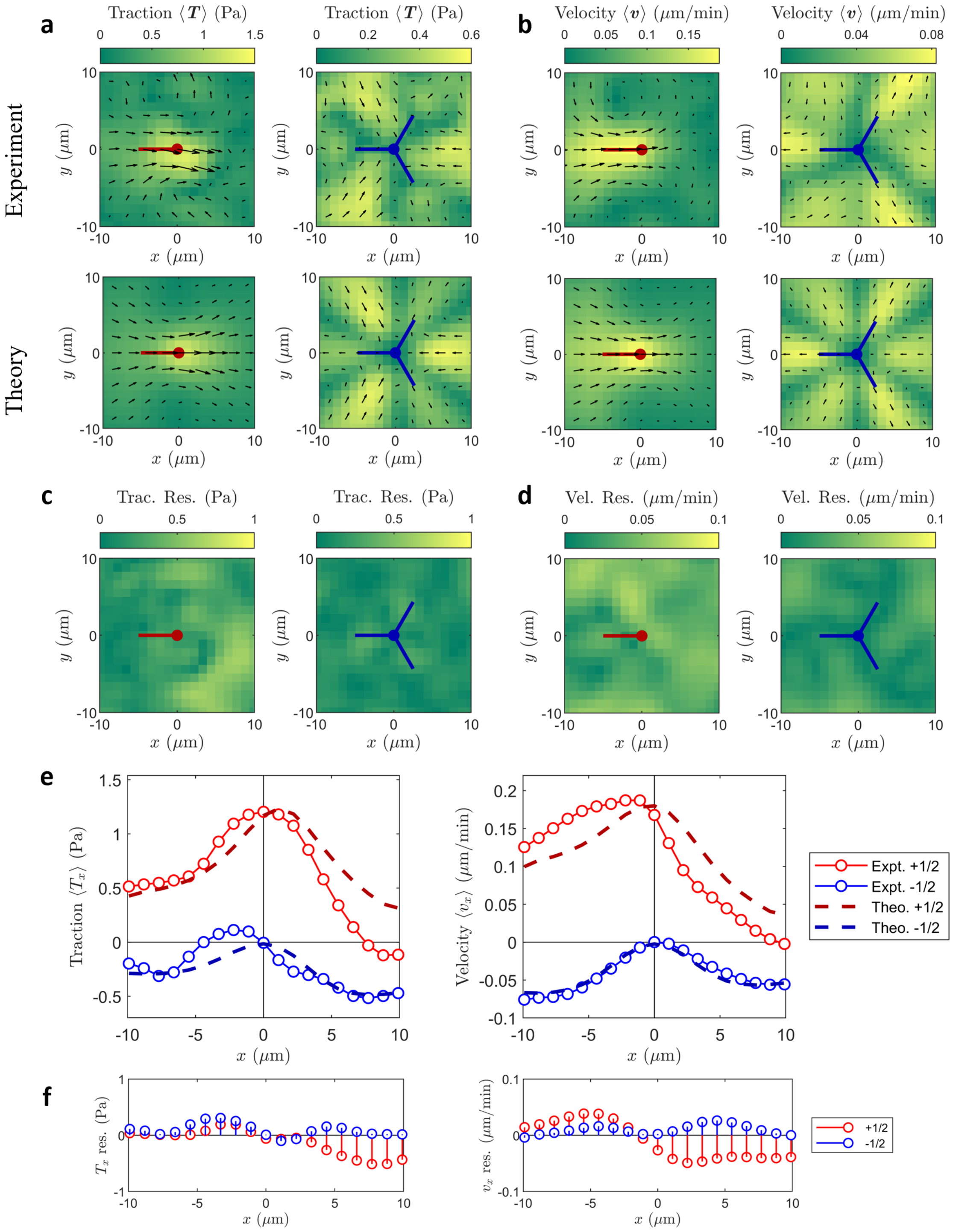

Fig. S1: Comparison between experimentally measured mean traction $\langle \boldsymbol{T} \rangle$ and mean velocity $\langle \boldsymbol{v} \rangle$, and the corresponding theoretical predictions. (a) Mean traction $\langle \boldsymbol{T} \rangle$ near $+1/2$ (red symbols) and $-1/2$ (blue symbols) defects. (b) Mean velocity of the cell flow $\langle \boldsymbol{v} \rangle$ near $+1/2$ (red symbols) and $-1/2$ (blue symbols) defects. In both panels, the top row is experimental and the bottom row is theoretical. The experimental measurements were obtained with the TFM assay (SI Sec. VIII). Both $\langle \boldsymbol{v} \rangle$ and $\langle \boldsymbol{T} \rangle$ were calculated with 7354 frames of $+1/2$ defects and 6640 frames of $-1/2$ defects identified in 11 replicated experiments. Details on how the theoretical velocity and traction distributions were calculated are in Sec. II. (c,d) Distributions of residuals in traction $|\boldsymbol{T}_{\rm expt} - \boldsymbol{T}_{\rm theo}|$ (c) and velocity $|\boldsymbol{v}_{\rm expt} - \boldsymbol{v}_{\rm theo}|$ (d) around defects. (e) Compare experimental (circles) and theoretical (dashed lines) $\langle T_x(x) \rangle$ and $\langle v_x(x) \rangle$ at $y = 0$ μm near the $+1/2$ (red) and $-1/2$ (blue) defects. The experimental data shown here are calculated with 7354 frames for the $+1/2$ defects and 6640 frames for the $-1/2$ defects. These defects are identified and tracked in 11 replicated experiments. (f) Residuals in T_x and v_x, $T_{x,\rm expt} - T_{x,\rm theo}$ and $v_{x,\rm expt} - v_{x,\rm theo}$, respectively.

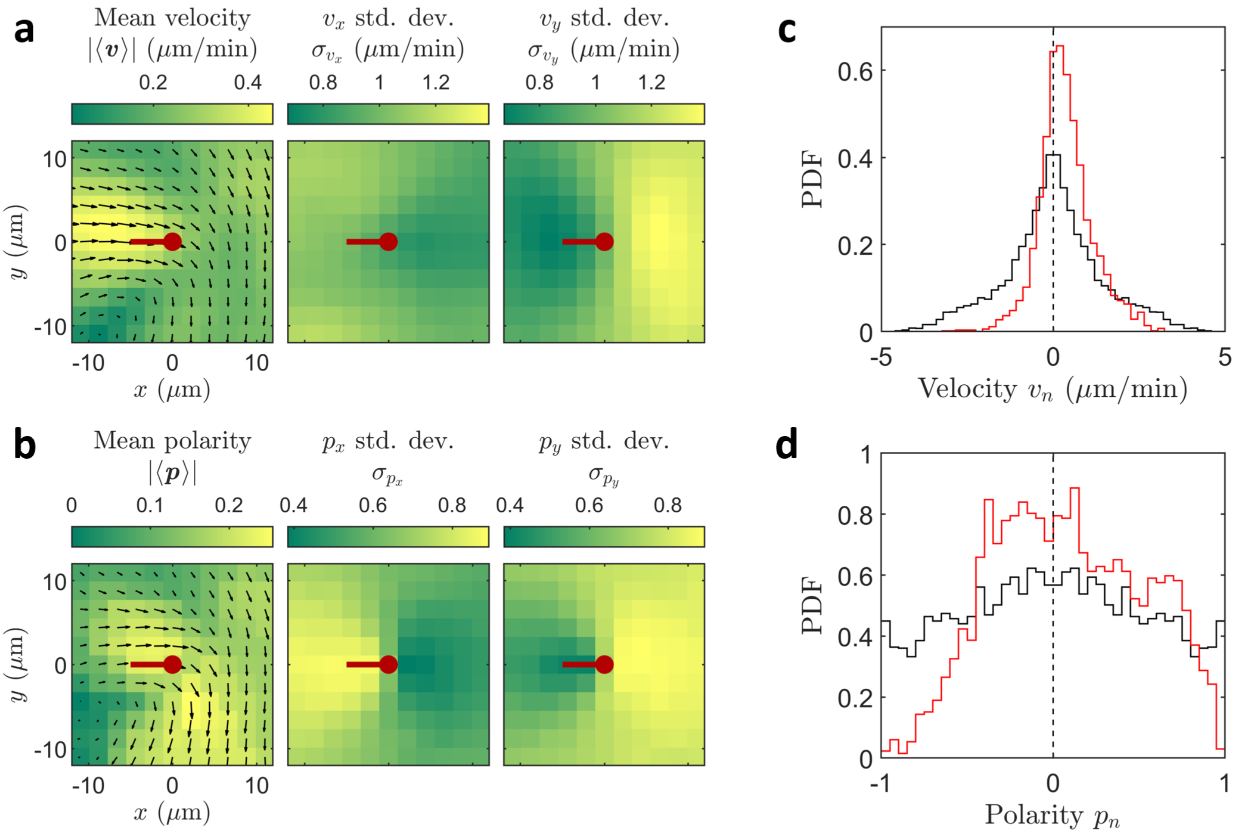

Fig. S2: Polarity and velocity in ordered regions and near +1/2 defects obtained with the polarity assay. (a) Mean velocity of cell flow $\langle \boldsymbol{v} \rangle$ and standard deviations of v_x and v_y (σ_{v_x} and σ_{v_y}) near +1/2 defects. The black arrows show the magnitude and direction of $\langle \boldsymbol{v} \rangle$. The color maps show $|\langle \boldsymbol{v} \rangle|$, σ_{v_x}, and σ_{v_y}, respectively. Here $\langle\ \rangle$ denotes the temporal average across all the frames in the comoving frame of the defect. (b) Mean cell polarity $\langle \boldsymbol{p} \rangle$ and standard deviations of p_x and p_y (σ_{p_x} and σ_{p_y}) near +1/2 defects. The black arrows show the magnitude and direction of $\langle \boldsymbol{p} \rangle$. The color maps show $|\langle \boldsymbol{p} \rangle|$, σ_{p_x}, and σ_{p_y}, respectively. (c) Probability density functions (PDF) of v_n in regions with aligned cells (black) and in the tail region of +1/2 defects (red). (d) PDF of p_n in regions with aligned cells (black) and in the tail region of +1/2 defects (red). In (c) and (d), v_n and p_n are calculated as described in SI Sec. V. The positive and negative directions are defined as shown by the orange arrows in Fig. S19.

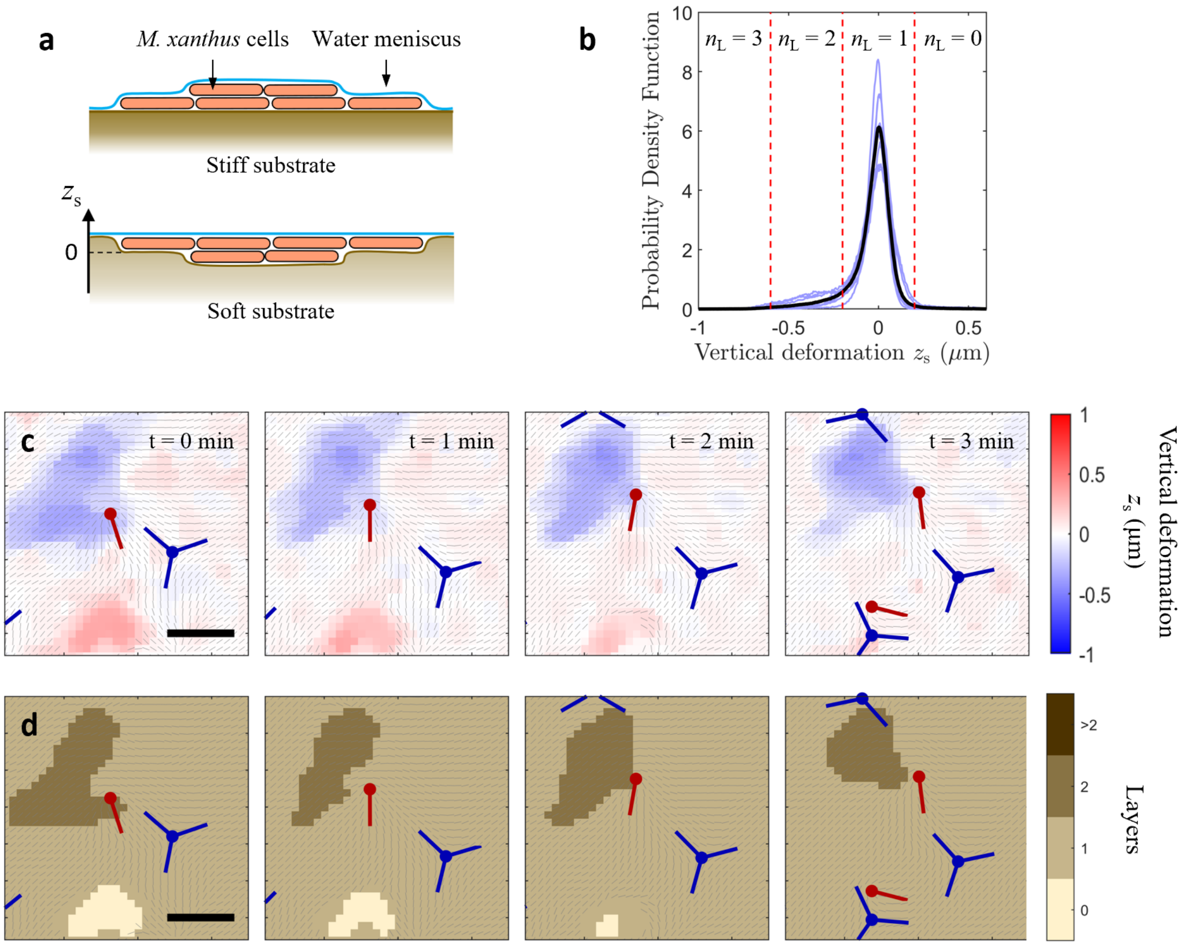

Fig. S3: Layer thickness obtained from the measurement of surface deformation z_s. The way we measured z_s is discussed in SI Sec. VII. (a) Illustration of *M. xanthus* layers on a rigid (top) and soft (bottom) substrate. The orange rods represent the cells, the cyan lines are air-water interfaces, and the brown lines represent the surfaces of the solid substrates. On a solid substrate, the water-air interface is deformed to conform to the shape of the cell layer. However, on a soft substrate, such as in our TFM assay, the substrate was so soft that deforming the substrate was easier than deforming the water-air interface. As a result, the cells were actually mostly embedded into the gel. We define $z_s = 0$ μm as the position just below a cell monolayer, so a double layer has $z_s < 0$ and regions on the substrate without any cell have $z_s > 0$. (b) Probability distribution function (PDF) of z_s obtained with Δ*pilA* cells and the TFM assay. Each blue curve was obtained from a video in the experiments. The black curve shows the PDF of all the data. The thickness of each cell layer was about 0.4 μm, thus we chose the red dashed lines as thresholds that turned z_s into integer cell layer thickness n_L. (c) Exemplary surface deformation z_s measured with the TFM assay. The color map shows z_s and the gray lines show the local director field $\hat{\boldsymbol{n}}$. The scale bar is 10 μm. (d) Layer thickness of the cell colony n_L based on z_s. The color map shows n_L and the gray lines show the local director field $\hat{\boldsymbol{n}}$. The scale bar is 10 μm.

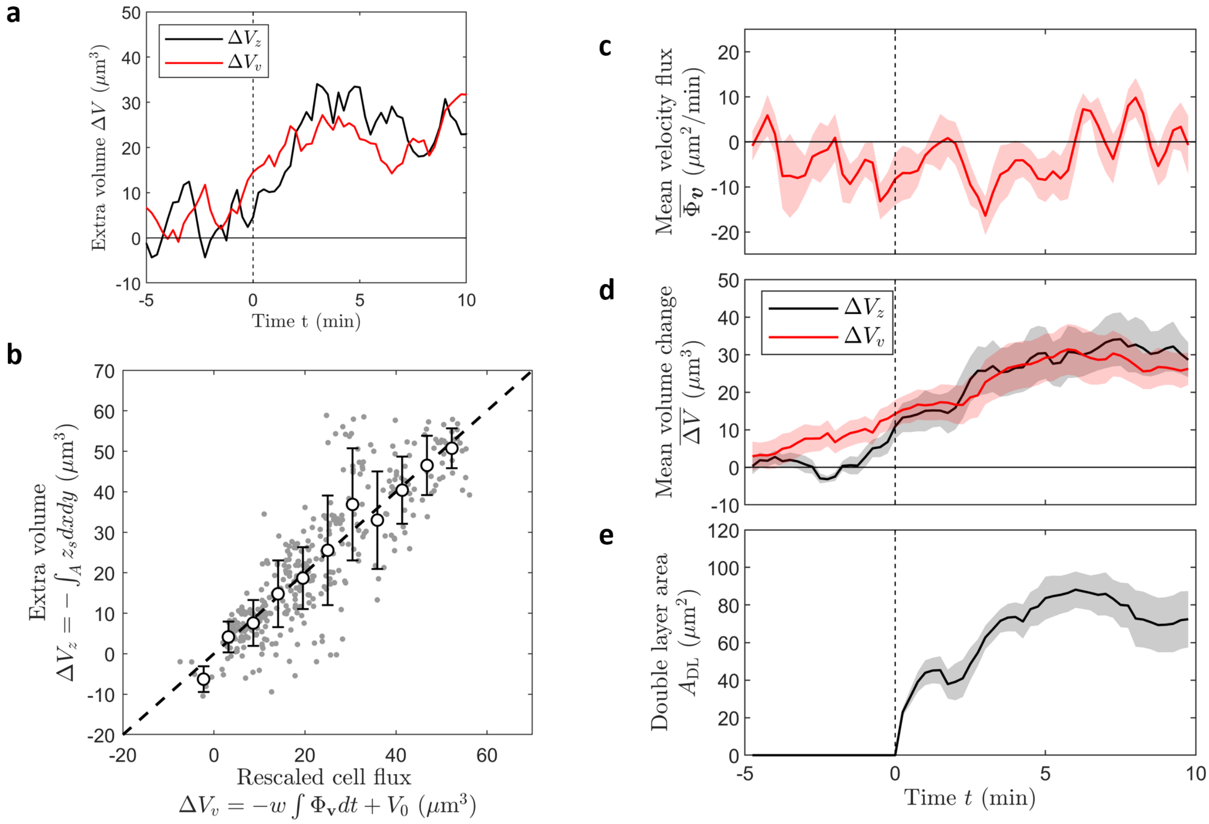

Fig. S4: Equivalence and difference of locally accumulated colony volume obtained with substrate surface deformation z_{s} and cell velocity $\boldsymbol{v}$. The minus surface deformation $-z_{\mathrm{s}}$ is equivalent to the thickness of the cell layer h: $-z_{\mathrm{s}} = h$. (a) An exemplary double-layer formation event where the local colony volume increased. The extra volume ΔV was calculated in two ways: (1) using z_{s}, $\Delta V_z = -\int_A z_{\mathrm{s}} \mathrm{d}x\mathrm{d}y$, where A is the area of a circular region of interest with a radius of $l_p = 12$ μm; and (2) using $\boldsymbol{v}$, $\Delta V_v = -w \int \Phi_v \mathrm{d}t + V_0$, where w is the thickness of the colony at the boundary of this circular region $\mathcal{C}$, $\Phi_v = \oint_{\mathcal{C}} (\boldsymbol{v} \cdot \hat{\boldsymbol{r}})\, ds$ is the cell flux out of this region, and V_0 represents $\Delta V_v(t = 0)$. We calculated ΔV_z and $\int \Phi_v \mathrm{d}t$ directly, and used w and V_0 as fitting parameters to match ΔV_z and ΔV_v. The time $t = 0$ min was when a double cell layer appeared. (b) Direct comparison between ΔV_z and ΔV_v. The gray data points are from seven different regions like what (a) shows. The black circles show the mean and the error bars show the standard deviation. The black dashed line is $\Delta V_z = \Delta V_v$. The layer thickness obtained was $\overline{w} = 0.46 \pm 0.18$ μm, which agrees with the thickness of one cell layer. (c) Mean cell flux $\overline{\Phi_v}$ (red line) and its standard error (shaded area) calculated using the same seven regions as in (b). (d) Volume change ΔV_z (red) and ΔV_v (black). The solid lines show the mean and the shaded areas show the corresponding standard errors. (e) Area of the double layer A_{DL}. The solid line shows the mean and the shaded area show the corresponding standard error. A double layer was defined as regions with $z_{\mathrm{s}} < -0.2$ μm. The time $t = 0$ min was when a double layer appeared in panels (c), (d), and (e). We can see that ΔV_z started to increase before a visible double layer actually appeared, because of the threshold in defining double layers. More interestingly, ΔV_v started increasing even earlier, which we think is due to the compression of cell colony locally. In other words, the local cell concentration increased first due to the cell influx, then the surface of the substrate started showing visible deformation, and eventually a visible double layer formed.

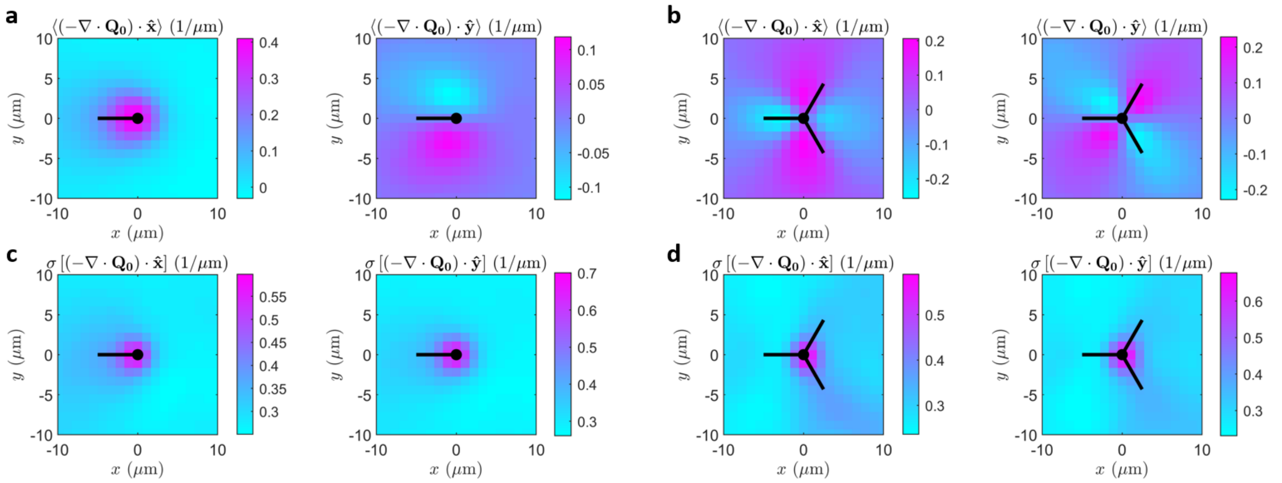

Fig. S5: Mean and fluctuation of $-\nabla \cdot \boldsymbol{Q}_0$, where $\boldsymbol{Q}_0 = \begin{pmatrix} \cos(2\langle\theta\rangle) & \sin(2\langle\theta\rangle) \\ \sin(2\langle\theta\rangle) & -\cos(2\langle\theta\rangle) \end{pmatrix}$ and $\langle\theta\rangle$ is the local average director angle. (a) Mean components of $-\nabla\cdot\boldsymbol{Q}_0$ near $+1/2$ defects in the x and y directions, $\langle(-\nabla\cdot\boldsymbol{Q}_0)\cdot\hat{\boldsymbol{x}}\rangle$ and $\langle(-\nabla\cdot\boldsymbol{Q}_0)\cdot\hat{\boldsymbol{y}}\rangle$, where $\hat{\boldsymbol{x}}$ and $\hat{\boldsymbol{y}}$ are unit vectors in the x and y directions, respectively. (b) Mean x and y components of $-\nabla\cdot\boldsymbol{Q}_0$ near $-1/2$ defects, $\langle(-\nabla\cdot\boldsymbol{Q}_0)\cdot\hat{\boldsymbol{x}}\rangle$ and $\langle(-\nabla\cdot\boldsymbol{Q}_0)\cdot\hat{\boldsymbol{y}}\rangle$. (c) Standard deviations of the x and y components of $-\nabla\cdot\boldsymbol{Q}_0$ near $+1/2$ defects, $\sigma\,[(-\nabla\cdot\boldsymbol{Q}_0)\cdot\hat{\boldsymbol{x}}]$ and $\sigma\,[(-\nabla\cdot\boldsymbol{Q}_0)\cdot\hat{\boldsymbol{y}}]$. (d) Standard deviations of the x and y components of $-\nabla\cdot\boldsymbol{Q}_0$ near $-1/2$ defects, $\sigma\,[(-\nabla\cdot\boldsymbol{Q}_0)\cdot\hat{\boldsymbol{x}}]$ and $\sigma\,[(-\nabla\cdot\boldsymbol{Q}_0)\cdot\hat{\boldsymbol{y}}]$. Within all the components of $\langle-\nabla\cdot\boldsymbol{Q}_0\rangle$ near $\pm1/2$ defects, the most significant one was $\langle(-\nabla\cdot\boldsymbol{Q}_0)\cdot\hat{\boldsymbol{x}}\rangle$ for $+1/2$ defects. All the standard deviations in (c) and (d) are of the same order of magnitude. As a result, the fluctuation in the director field alone does not explain the huge fluctuations in the velocity and mechanical stress in the system, which were both about one order of magnitude stronger than the mean.

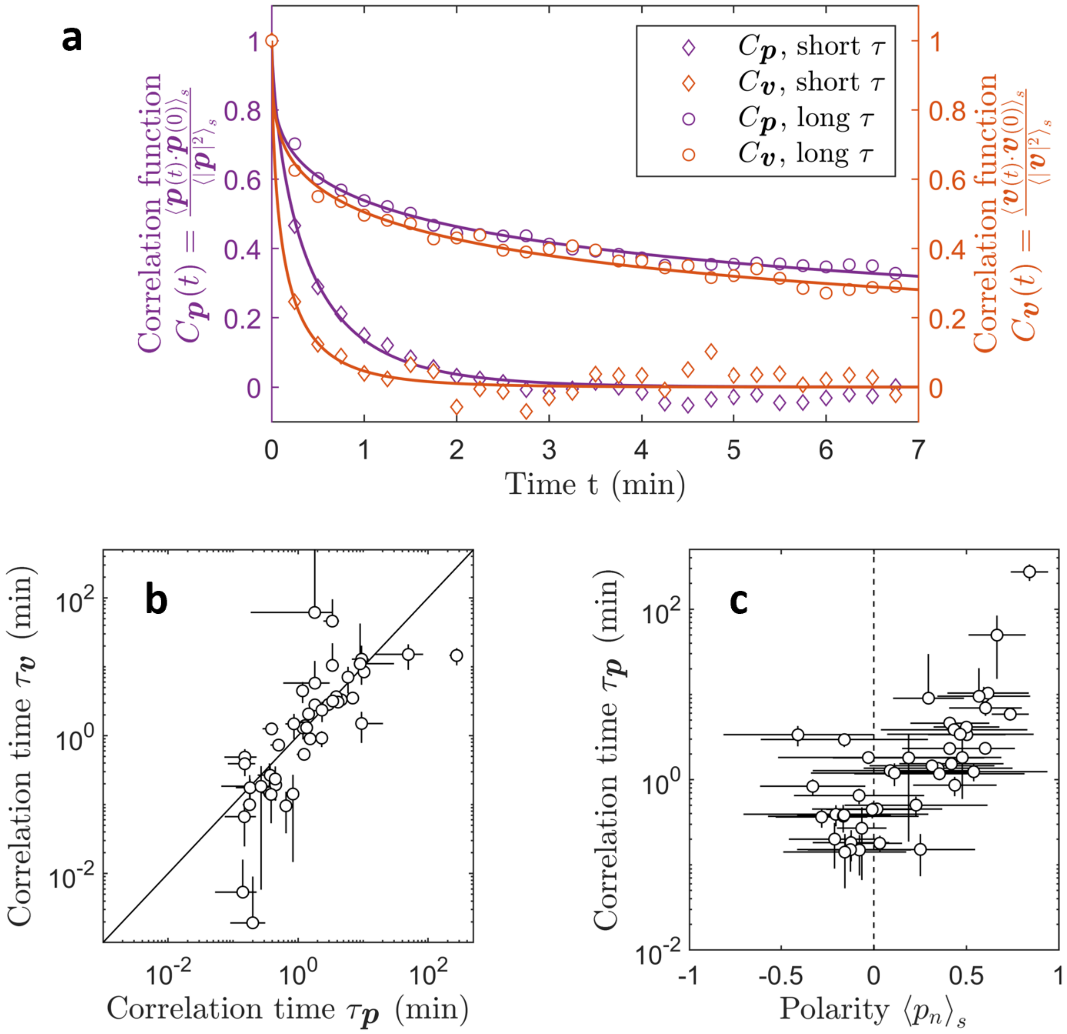

Fig. S6: Correlation functions and correlation times of polarity $\boldsymbol{p}$ and velocity $\boldsymbol{v}$ in the tail regions of $+1/2$ defects. (a) Exemplary correlation functions of polarity $C_p(t) = \frac{\langle \boldsymbol{p}(t)\cdot\boldsymbol{p}(0)\rangle_\mathrm{s}}{\langle |\boldsymbol{p}|^2\rangle_\mathrm{s}}$ and velocity $C_v(t) = \frac{\langle \boldsymbol{v}(t)\cdot\boldsymbol{v}(0)\rangle_\mathrm{s}}{\langle |\boldsymbol{v}|^2\rangle_\mathrm{s}}$. The data were fitted with a stretched exponential function $C(t) = \exp\left[-(t/\tau)^\beta\right]$, where τ is the correlation time and β is a fitting parameter. A pair of $C_p(t)$ and $C_v(t)$ obtained from the data of one defect with relatively short τ (diamonds) and another pair from another defect with relatively long τ (circles) are shown in the plot. The solid curves show the corresponding best fits. (b) Compare correlation times obtained with polarity $\tau_{\boldsymbol{p}}$ and velocity $\tau_{\boldsymbol{v}}$. They were approximately equivalent across two orders of magnitude. (c) Relationship between the correlation time $\tau_{\boldsymbol{p}}$ and the average local polarity $\langle p_n\rangle_\mathrm{s}$. Higher polarity leads to a longer correlation time. Each data point in (b) and (c) corresponds to one video of a $+1/2$ defect.

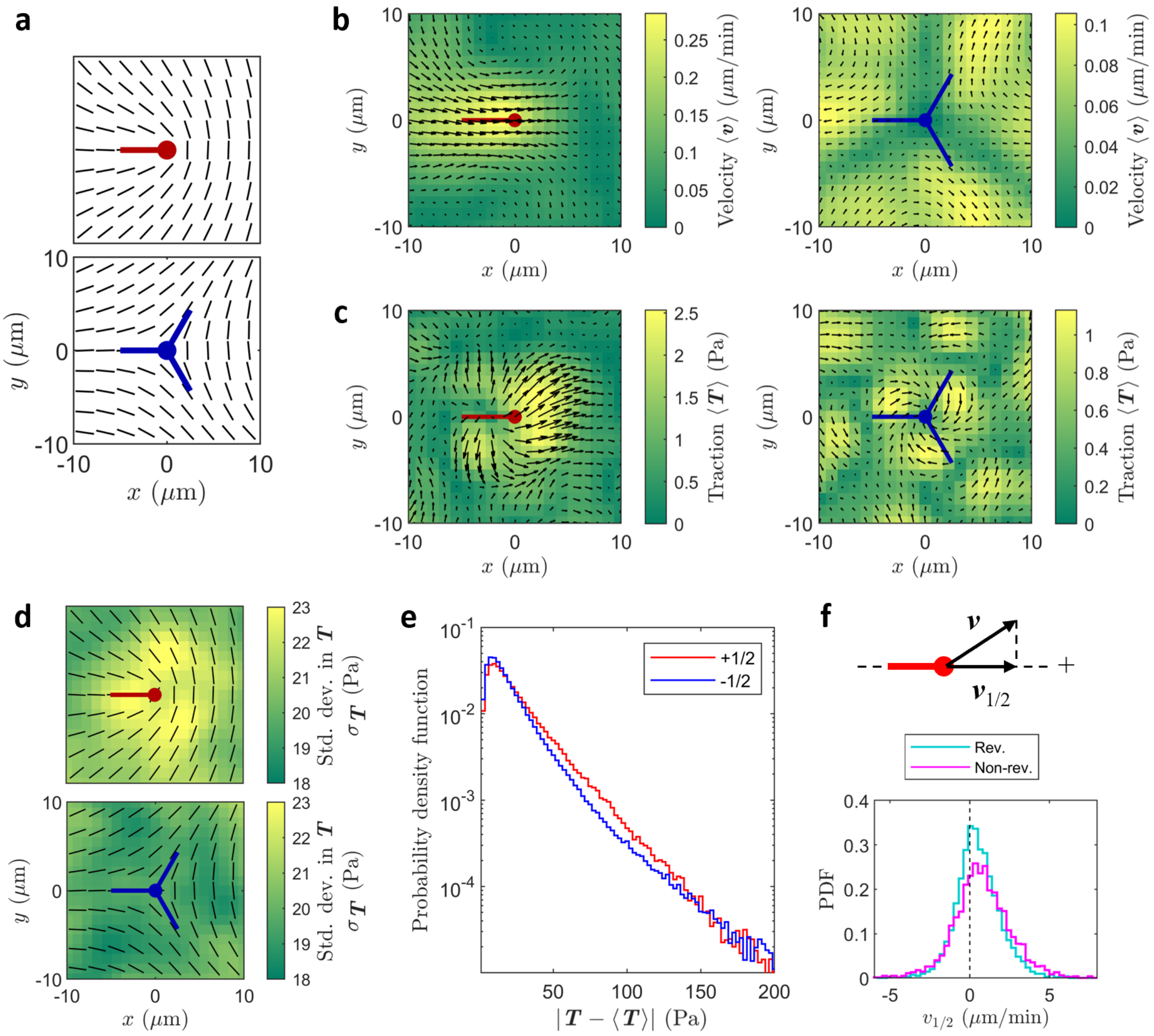

Fig. S7: Properties of the $\pm 1/2$ defects in thin colonies of $\Delta frzE$ cells (without reversal). (a) Experimentally measured mean directors $\langle \hat{\boldsymbol{n}} \rangle$ near the $\pm 1/2$ defects. (b) Experimentally measured mean velocity of cell flow $\langle \boldsymbol{v} \rangle$ near the $\pm 1/2$ defect. The black arrows show its magnitude and direction and the color map shows the speed $|\langle \boldsymbol{v} \rangle|$. (c) Experimentally measured mean traction $\langle \boldsymbol{T} \rangle$ near $\pm 1/2$ defects. The color maps show their magnitudes, and the arrows label their magnitude and direction. (d) Experimentally measured standard deviation of traction $\sigma_{\boldsymbol{T}}$ near $\pm 1/2$ defects. The black lines show $\langle \hat{\boldsymbol{n}} \rangle$. (e) Distributions of traction fluctuations $|\boldsymbol{T} - \langle \boldsymbol{T} \rangle|$ within 5 μm from the centers of $+1/2$ (red) and $-1/2$ (blue) defects. (f) Distributions of the forward moving velocity of $+1/2$ defects $v_{1/2}$ for the $\Delta pilA$ (reversing) and $\Delta frzE$ (non-reversing) cells. The sketch above shows how we defined the defect velocity $v_{1/2}$. The right-hand side is the positive direction.

II. THEORIES ON TRACTION AND CELL FLOW NEAR DEFECTS

A. Landau-de Gennes theory

We use the Landau-de Gennes theory to describe the nematic state of dense, planar bacterial colonies. The tensorial order parameter is defined as

$$\boldsymbol{Q} = 2S\Big(\hat{\boldsymbol{n}}\hat{\boldsymbol{n}} - \frac{1}{2}\boldsymbol{I}\Big), \tag{S1}$$

where the director $\hat{\boldsymbol{n}} \equiv (\cos\theta, \sin\theta)$ denotes the local axis along which cells align, $\boldsymbol{I}$ is the unit matrix, and S is the scalar order parameter. The 2D Laudau-de Gennes energy functional takes the form

$$\mathcal{F}(\boldsymbol{Q}, \nabla\boldsymbol{Q}) = \int d^2\boldsymbol{x}\Big[-\frac{a}{2}\mathrm{Tr}(\boldsymbol{Q}^T\boldsymbol{Q}) + \frac{b}{4}\Big(\mathrm{Tr}(\boldsymbol{Q}^T\boldsymbol{Q})\Big)^2 + \frac{L}{2}(\nabla\boldsymbol{Q}) : (\nabla\boldsymbol{Q})\Big], \tag{S2}$$

where $a, b > 0$ and L denotes the orientation elastic modulus. Note that here we do not include the $\partial_j Q_{ij}\partial_k Q_{ik}$ invariant because in 2D there is no twisting mode of the distortion, which corresponds to the Frank elastic energy $\frac{K_3}{2}|\hat{\boldsymbol{n}} \times \nabla \times \hat{\boldsymbol{n}}|^2$.

Introducing Eq. S1 into Eq. S2, we obtain the following nematic energy:

$$\mathcal{F}(S, \theta, \nabla S, \nabla\theta) = \int d^2\boldsymbol{x}\Big[-aS^2 + bS^4 + L|\nabla S|^2 + 4LS^2|\nabla\theta|^2\Big]. \tag{S3}$$

In the uniformly ordered phase, the order parameter strength is given by $S_0^2 = a/2b$. Around a defect of charge q, where $\theta = q\phi$, and $S = S_0\Phi(r)$, we obtain $\nabla\theta = q/r\ \boldsymbol{e}_\phi$ and $\nabla S = S_0\Phi'(r)\ \boldsymbol{e}_r$. Introducing these relations into Eq. S3, we obtain, for $\pm1/2$ defects ($q = \pm1/2$),

$$\mathcal{F}[\Phi] = LS_0^2\int 2\pi\tilde{r}d\tilde{r}\Big[-\Phi^2 + \frac{1}{2}\Phi^4 + \Big(\frac{d\Phi}{d\tilde{r}}\Big)^2 + \frac{\Phi^2}{\tilde{r}^2}\Big], \tag{S4}$$

where we defined a dimensionless radial coordinate as $\tilde{r} = r/\ell$, in which $\ell = \sqrt{L/a}$ denotes the nematic correlation length. Minimizing the energy functional yields the saturating coefficient of the nematic order $\Phi(\tilde{r})$, which satisfies $\Phi(0) = 0$ and $\Phi(\infty) = 1$ and the ODE,

$$-\Phi + \Phi^3 = \Phi'' + \Phi'/\tilde{r} - \Phi/\tilde{r}^2, \tag{S5}$$

where Φ' denotes $d\Phi/d\tilde{r}$. Although the exact expression for Φ cannot be solved analytically, it can be approximated by the Padé approximant:

$$\Phi(\tilde{r}) \approx \sqrt{\frac{0.07\tilde{r}^4 + 0.34\tilde{r}^2}{1 + 0.41\tilde{r}^2 + 0.07\tilde{r}^4}}. \tag{S6}$$

The alignment order parameter S measured by experiments can be fitted by

$$S(r) = S_0\Phi(r/\ell)\exp(-r/R), \tag{S7}$$

where S_0, ℓ, and R are fitting parameters, and Φ is given by Eq. S6. The exponential decay is purely phenomenological. In each frame, in the regions away from the defect cores, the cells are normally well aligned. However, in different frames (at different times), they may align in different directions. Consequently, when averaged across all frames, the mean orientational order is weak away from the defect cores. This is why we introduce an exponential term here in Eq. S7. The measured $S(r)$ are similar between $+1/2$ defects and $-1/2$ defects (Fig. S8). Thus, we use the same parameters to fit both data, yielding $S_0 = 1.2$, $\ell = 0.8\ \mu m$, and $R = 10\ \mu$m. Note that as a fitting parameter, S_0 is allowed to exceed 1, while the order parameters S must be in the range of $[0, 1]$. For regions around the topological defects, the complete form of $\boldsymbol{Q}$ is given by

$$\boldsymbol{Q}(r, \phi) = S(r)\begin{pmatrix}\cos(2q\phi) & \sin(2q\phi)\\ \sin(2q\phi) & -\cos(2q\phi)\end{pmatrix}, \tag{S8}$$

where $S(r)$ is given by Eq. S7.

B. Hydrodynamics of two-dimensional incompressible cell monolayers

Here, we consider the flow in a two-dimensional nematic system driven by the active stress and dampened by drag-like friction between cells and substrate. In such a microscopic system, inertia plays no role, so the force balance equation is

$$\boldsymbol{f}_\mathrm{s}^\mathrm{a} + \boldsymbol{f}_\mathrm{c}^\mathrm{a} + \boldsymbol{f}_\mathrm{s}^\mathrm{f} + \boldsymbol{f}_\mathrm{c}^\mathrm{f} - \nabla P + \nabla\cdot\boldsymbol{\sigma}^\mathrm{el} + \nabla\cdot\boldsymbol{\sigma}^\mu = 0. \tag{S9}$$

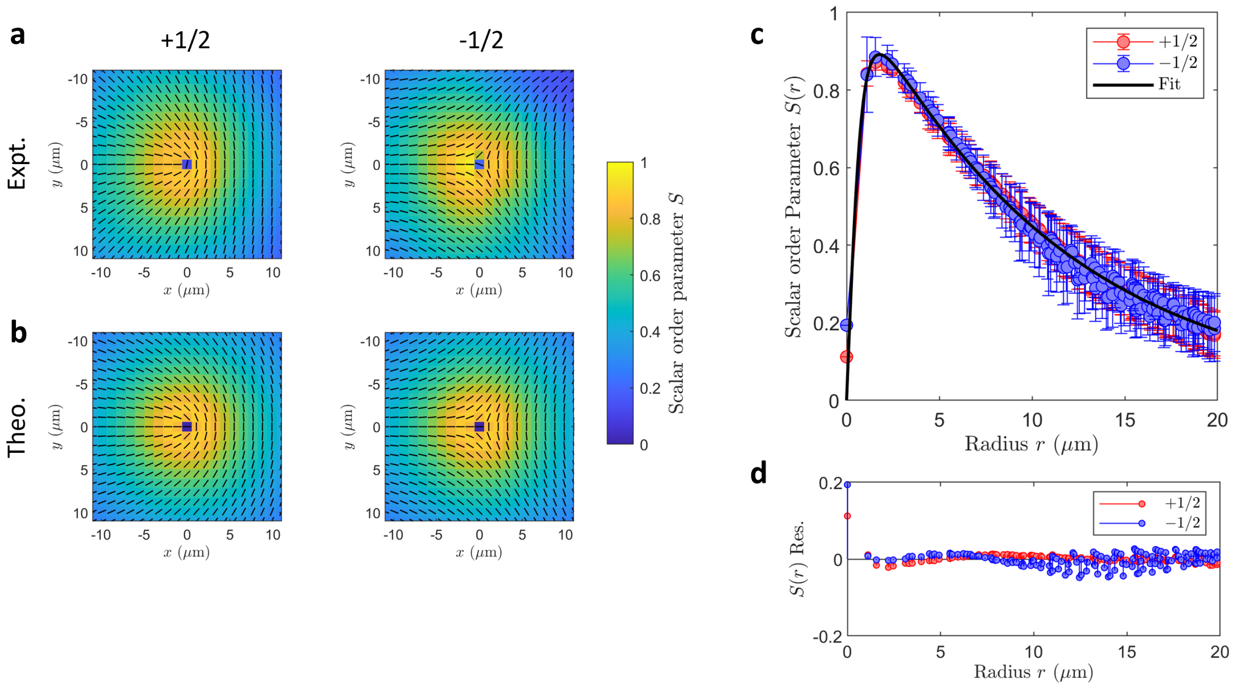

Fig. S8: Director field and scalar order parameter S around $\pm 1/2$ defects. (a) Experimentally measured mean director fields around $+1/2$ (left) and $-1/2$ (right) defects. The color map shows the scalar order parameter S and the black lines label the local directors. (b) Theoretically calculated director fields around $+1/2$ (left) and $-1/2$ (right) defects. Labels identical to (a). (c) Radial distribution of the scalar order parameter S for $+1/2$ (red) and $-1/2$ (blue) defects. The black curve is the best fit using Eq. S7. (d) Residuals in $S(r)$ around +1/2 and -1/2 defects, $S_{\rm expt}(r) - S_{\rm fit}(r)$.

Term	Description
$\boldsymbol{f}_{\rm s}^{\rm a}$	Active force density due to cells gliding on substrate
$\boldsymbol{f}_{\rm c}^{\rm a}$	Active force density due to cells gliding on other cells
$\boldsymbol{f}_{\rm s}^{\rm f}$	Density of friction between cells and substrate
$\boldsymbol{f}_{\rm c}^{\rm f}$	Density of friction between cells
∇P	Pressure gradient
$\nabla \cdot \boldsymbol{\sigma}^{\rm el}$	Force density arising from nematic elasticity
$\nabla \cdot \boldsymbol{\sigma}^{\mu}$	Viscous force density

TABLE S1: Different force densities in the system. Their units are all Pa. The pressure P and stresses $\sigma^{\rm el}$ and σ^{μ} are all linear force densities with the unit Pa·m.

The meanings of the terms are described in Table S1. The basic idea is that cells self-propel by exerting active forces both on the substrate $\boldsymbol{f}_{\rm s}^{\rm a}$ and on other cells $\boldsymbol{f}_{\rm c}^{\rm a}$, and they experience frictions applied by the substrate $\boldsymbol{f}_{\rm s}^{\rm f}$ and by other cells $\boldsymbol{f}_{\rm c}^{\rm f}$. The pressure P ensures that the velocity field $\boldsymbol{v}$ satisfies any imposed velocity-divergence conditions. Lastly, there is an elastic term $\nabla \cdot \boldsymbol{\sigma}^{\rm el}$ due to distortions of the nematic director, and a viscous term $\nabla \cdot \boldsymbol{\sigma}^{\mu}$ due to cell-cell friction. Following previous work [1], we neglected these last two terms in our calculation.

What is the traction $\boldsymbol{T}$ measured in our TFM experiments? It reflects the total force applied on the substrate by the cells, thus corresponding to the sum of cell-substrate forces, $\boldsymbol{f}_{\rm s}^{\rm a} + \boldsymbol{f}_{\rm s}^{\rm f}$. By virtue of the force balance Eq. S9, the traction can also be expressed as the sum of the internal forces in the cell layer:

$$\boldsymbol{T} = -\boldsymbol{f}_{\rm s}^{\rm a} - \boldsymbol{f}_{\rm s}^{\rm f}, \tag{S10a}$$

$$\boldsymbol{T} = \boldsymbol{f}_{\rm c}^{\rm a} + \boldsymbol{f}_{\rm c}^{\rm f} - \nabla P. \tag{S10b}$$

The active force due to cells gliding on the substrate, $\boldsymbol{f}_s^{\rm a}$ is controlled by the instantaneous polarity $\boldsymbol{p}$ of the cells: each cell pushes the substrate in the direction opposite to its polarity, so we can write

$$\boldsymbol{f}_{\rm s}^{\rm a} = \zeta_p \boldsymbol{p}. \tag{S11}$$

Following [1], we model the friction force as an anisotropic viscous force that depends on the orientation field of the bacterial cells:

$$\boldsymbol{f}_{\rm s}^{\rm f} = -\xi_0 (\boldsymbol{I} - \epsilon \boldsymbol{Q}) \cdot \boldsymbol{v}, \tag{S12}$$

where ξ_0 is the isotropic contribution to the friction coefficient, ϵ is the friction anisotropy, and $\boldsymbol{v}$ denotes the velocity of the cells. The cell-cell active force $\boldsymbol{f}_{\rm c}^{\rm a}$ results from force dipoles generated between cell pairs. This

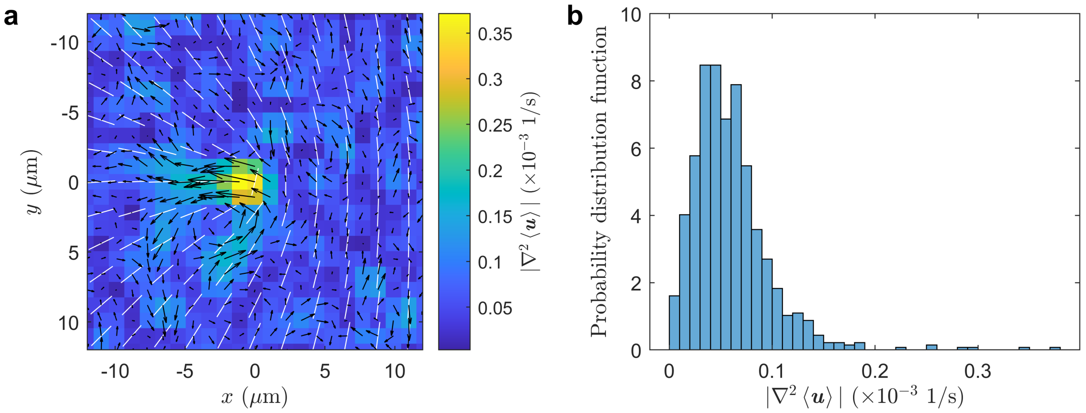

Fig. S9: Distribution of $\nabla^2 \langle \boldsymbol{u} \rangle$ near a +1/2 defect. (a) The color map shows the magnitude of $\left|\nabla^2 \langle \boldsymbol{u} \rangle\right|$. The black arrows indicate the magnitude and direction of $\nabla^2 \langle \boldsymbol{u} \rangle$. The white bars show the average director field. (b) Distribution of $\left|\nabla^2 \langle \boldsymbol{u} \rangle\right|$ in (a).

force is, therefore, the standard active force in active nematics, which emerges from the active stress $\boldsymbol{\sigma}_{\mathrm{c}}^{\mathrm{a}} = \zeta_{\mathrm{c}} \boldsymbol{Q}$, with $\boldsymbol{f}_{\mathrm{c}}^{\mathrm{a}} = -\nabla \cdot \boldsymbol{\sigma}_{\mathrm{c}}^{\mathrm{a}}$, so we get

$$\boldsymbol{f}_{\mathrm{c}}^{\mathrm{a}} = -\zeta_{\mathrm{c}} \nabla \cdot \boldsymbol{Q}. \tag{S13}$$

Our system is described by extensile active stresses, with a positive active-stress coefficient $\zeta_{\mathrm{c}} > 0$. Lastly, $\boldsymbol{f}_{\mathrm{c}}^{\mathrm{f}}$ is controlled by the relative velocity between adjacent cells, and it is similar to the viscous dissipation term in the Navier-Stokes equations:

$$\boldsymbol{f}_{\mathrm{c}}^{\mathrm{f}} = h \mu_{\mathrm{c}} \nabla^2 \boldsymbol{v}, \tag{S14}$$

where h is the thickness of the cell layer and μ_{c} is the effective viscosity of the cell colony.

In our experiments, we measured the average traction near the defects. According to Eq. S10, we have

$$\langle \boldsymbol{T} \rangle = -\langle \boldsymbol{f}_{\mathrm{s}}^{\mathrm{a}} \rangle - \left\langle \boldsymbol{f}_{\mathrm{s}}^{\mathrm{f}} \right\rangle, \tag{S15a}$$

$$\langle \boldsymbol{T} \rangle = \langle \boldsymbol{f}_{\mathrm{c}}^{\mathrm{a}} \rangle + \left\langle \boldsymbol{f}_{\mathrm{c}}^{\mathrm{f}} \right\rangle - \langle \nabla P \rangle. \tag{S15b}$$

Within all these terms, $\left\langle \boldsymbol{f}_{\mathrm{c}}^{\mathrm{f}} \right\rangle$ is negligible. Given Eq. S14, we obtain

$$\left\langle \boldsymbol{f}_{\mathrm{c}}^{\mathrm{f}} \right\rangle = h \left\langle \mu_{\mathrm{c}} \nabla^2 \boldsymbol{v} \right\rangle = h \mu_{\mathrm{c}} \nabla^2 \langle \boldsymbol{v} \rangle. \tag{S16}$$

The spatial distribution of $\nabla^2 \langle \boldsymbol{v} \rangle$ is shown in Fig. S9. Except for the small region very close to the core of the defect, $\left|\nabla^2 \langle \boldsymbol{v} \rangle\right| < 2 \times 10^{-4}$ s^{-1}. According to a recent measurement [2], the loss modulus G'' of a 12-hour *M. xanthus* fruiting body is $G'' \approx 0.2$ kPa at frequency $\omega = 0.1$ Hz and $G'' \approx 1$ kPa at frequency $\omega = 1$ Hz. Considering the cell speed of the order of microns per minute in our system, 1 Hz is in the high-frequency regime. As a result, we estimate that the upper limit of the dynamic viscosity in our thin cell layers is about $\mu_{\mathrm{c}} = G''/\omega \approx 1$ kPa·s, which is 10^6 times more viscous than water. Furthermore, as a fruiting body ages, its viscosity increases [2]. In our cell monolayer, which is at a much earlier stage than a 12-hour fruiting body, we expect an even smaller viscosity. Using $\mu_{\mathrm{c}} = 1$ kPa·s and $h = 0.5$ μm to estimate the stress due to cell-cell drag, we get

$$\left\langle \boldsymbol{f}_{\mathrm{c}}^{\mathrm{f}} \right\rangle < 0.1 \text{ Pa} \tag{S17}$$

in most regions around a +1/2 defect. The actual value of $\left\langle \boldsymbol{f}_{\mathrm{c}}^{\mathrm{f}} \right\rangle$ could be even smaller given the way we chose the parameters' values. Thus in the following analysis, we assume this term is negligible. Note that here we neglected the anisotropy in the cell layer, but the estimated order of magnitude should hold.

Now we can introduce all the other terms above to Eqs. S15:

$$\langle \boldsymbol{T} \rangle = -\zeta_p \langle \boldsymbol{p} \rangle + \xi_0 \left\langle (\boldsymbol{I} - \epsilon \boldsymbol{Q}) \cdot \boldsymbol{v} \right\rangle, \tag{S18a}$$

$$\langle \boldsymbol{T} \rangle = -\zeta_{\mathrm{c}} \nabla \cdot \langle \boldsymbol{Q} \rangle - \nabla \langle P \rangle. \tag{S18b}$$

In the experiments, we measured $\langle \boldsymbol{T} \rangle$, $\langle \boldsymbol{Q} \rangle$ (based on θ), and $\langle \boldsymbol{v} \rangle$. The unknown fields are $\langle \boldsymbol{p} \rangle$ and $\langle P \rangle$ and the unknown parameters are ζ_p, ξ_0, ϵ, and ζ_{c}. Note that all the force densities here are areal force densities, which

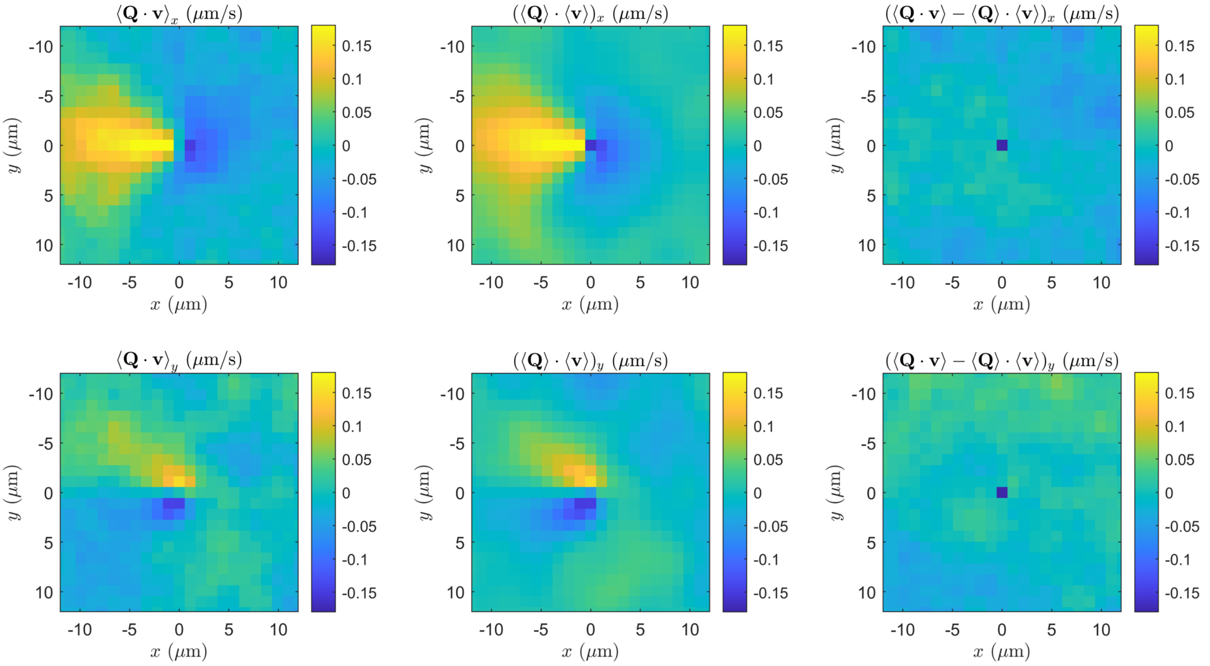

Fig. S10: The distributions of the x and y components of $\langle \boldsymbol{Q}\cdot\boldsymbol{v}\rangle$ (left column), $\langle \boldsymbol{Q}\rangle\cdot\langle\boldsymbol{v}\rangle$ (middle column), and $\langle \boldsymbol{Q}\cdot\boldsymbol{v}\rangle - \langle \boldsymbol{Q}\rangle\cdot\langle\boldsymbol{v}\rangle$ (right column).

have the unit of Pa, and the pressure P has the unit of Pa·m. Correspondingly, ζ_c has the unit of Pa·m, ζ_p has the unit of Pa, and ξ_0 has the unit of Pa·s/m.

We assume that the fluctuations in cell velocity $\langle\boldsymbol{v}\rangle$ and nematic order parameter $\langle\boldsymbol{Q}\rangle$ are uncorrelated, so

$$\langle \boldsymbol{Q} - \langle\boldsymbol{Q}\rangle\rangle \cdot \langle \boldsymbol{v} - \langle\boldsymbol{v}\rangle\rangle = \langle \boldsymbol{Q}\cdot\boldsymbol{v}\rangle - \langle\boldsymbol{Q}\rangle\cdot\langle\boldsymbol{v}\rangle = 0. \tag{S19}$$

This assumption makes sense for our system as a cell reversal event causes a velocity fluctuation without affecting the nematic order. Indeed, this assumption is confirmed by our experimental data in Fig. S10. As a result, $\langle \boldsymbol{Q}\cdot\boldsymbol{v}\rangle$ can be written as $\langle\boldsymbol{Q}\rangle\cdot\langle\boldsymbol{v}\rangle$, too, and Eq. S18a becomes

$$\langle\boldsymbol{T}\rangle = -\zeta_p\langle\boldsymbol{p}\rangle + \xi_0\langle(\boldsymbol{I}-\epsilon\boldsymbol{Q})\cdot\boldsymbol{v}\rangle = -\zeta_p\langle\boldsymbol{p}\rangle + \xi_0\langle\boldsymbol{I}-\epsilon\boldsymbol{Q}\rangle\cdot\langle\boldsymbol{v}\rangle\,. \tag{S20}$$

C. Velocity and traction around topological defects

Combining Eq. S18a and Eq. S18b and assuming mean polarity $\langle\boldsymbol{p}\rangle = 0$, the balance of average forces near defects is given by

$$\langle\boldsymbol{T}\rangle = \xi_0(\boldsymbol{I} - \epsilon\langle\boldsymbol{Q}\rangle)\cdot\langle\boldsymbol{v}\rangle = -\zeta_c\nabla\cdot\langle\boldsymbol{Q}\rangle - \nabla\langle P\rangle\,. \tag{S21}$$

The isotropic pressure $\langle P\rangle$ ensures that $\langle\boldsymbol{v}\rangle$ satisfies the constraint $\nabla\cdot\langle\boldsymbol{v}\rangle = j(\boldsymbol{r})$, where j is the flux of cells across cell layers. For $-1/2$ defects, we impose the incompressibility condition $j(\boldsymbol{r}) \equiv 0$. To capture the asymmetric cell flows around the $+1/2$ defects (Fig. S1), we set

$$j(\boldsymbol{r}) = \begin{cases} -j_0 & \text{for } r \leqslant R_0, \\ 0 & \text{for } r > R_0, \end{cases} \tag{S22}$$

where r denotes the distance from the defect center, and $j_0 = 6\times10^{-3}$ min^{-1} and $R_0 = 8.9$ μm are determined from experimental measurements (Fig. S11). Since ζ_c sets the scale for the stress/pressure, we rewrite Eq. S21 with scaled quantities $\tilde{P} = P/\zeta_c$ and $\tilde{\xi} = \xi_0/\zeta_c$, which yields

$$\tilde{\xi}(\boldsymbol{I} - \epsilon\langle\boldsymbol{Q}\rangle)\cdot\langle\boldsymbol{v}\rangle = -\nabla\cdot\langle\boldsymbol{Q}\rangle - \nabla\left\langle\tilde{P}\right\rangle. \tag{S23}$$

We solve Eq. S23 numerically to obtain the velocity field $\langle\boldsymbol{v}\rangle$ as described below. The traction field $\langle\boldsymbol{T}\rangle$ is then computed by $\langle\boldsymbol{T}\rangle = \xi_0(\boldsymbol{I}-\epsilon\langle\boldsymbol{Q}\rangle)\cdot\langle\boldsymbol{v}\rangle$.

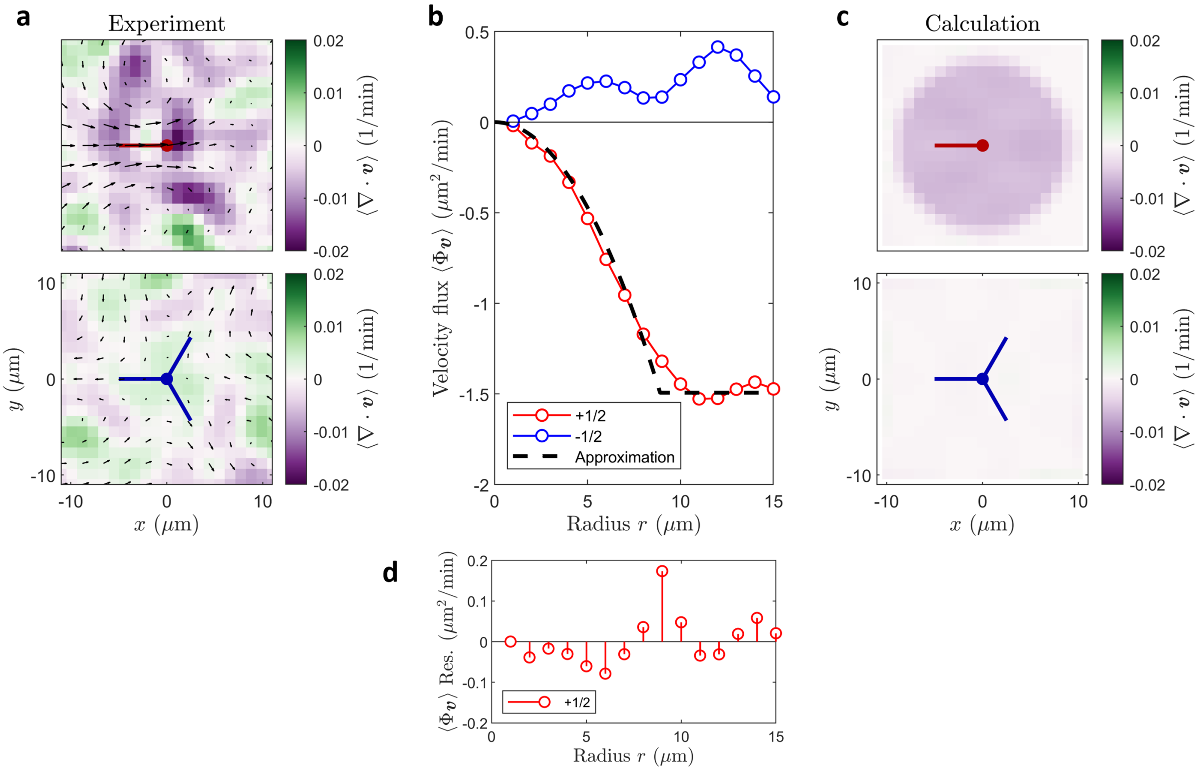

Fig. S11: Compressibility of the cell layer near $\pm 1/2$ defects. (a) Divergence of the mean cell velocity $\langle \nabla \cdot \boldsymbol{v} \rangle$ near $+1/2$ (red symbols) and $-1/2$ (blue symbols) defects. The color maps show $\langle \nabla \cdot \boldsymbol{v} \rangle$, with green being positive and purple being negative, and the black arrows show the magnitude and direction of local velocity $\langle \boldsymbol{v} \rangle$. The experimental measurements were obtained with the TFM assay (SI Sec. VIII). The mean velocities were calculated with 7354 frames of $+1/2$ defects and 6640 frames of $-1/2$ defects identified in 11 replicated experiments. We tracked the motion of these defects, aligned the velocity fields around them in every frame based on the defects' locations and orientations, and then calculated the mean. Note that even though the velocity fields were aligned in the comoving frame of the defects, what we show here and in the main text are the cell velocities with respect to the substrate (in the lab frame). (b) Velocity flux Φ_v across a circular region with radius r and centered at $+1/2$ (red) and $-1/2$ (blue) defects, $\Phi_v = \oint_{\mathcal{C}} (\boldsymbol{v} \cdot \hat{\boldsymbol{r}})\, \mathrm{d}s = \iint_{\mathcal{A}} (\nabla \cdot \boldsymbol{v})\, \mathrm{d}x\mathrm{d}y$, where $\mathcal{C}$ is the boundary of this circular region and $\mathcal{A}$ is the area it covers. The flux near the $+1/2$ defect was approximated by a parabolic function at small r ($r < 8.9$ μm) and a constant where $r > 8.9$ μm, as the black dashed line shows. This means that in the parabolic regime ($r < 8.9$ μm) $\langle \nabla \cdot \boldsymbol{v} \rangle$ is approximately a constant, and $\langle \nabla \cdot \boldsymbol{v} \rangle = 0$ as $r > 8.9$ μm (c) In the theoretical calculations, we used the approximated spatial distribution of $\langle \nabla \cdot \boldsymbol{v} \rangle$ as a constraint. We assumed $\langle \nabla \cdot \boldsymbol{v} \rangle =$ constant as $r < 8.9$ μm and 0 otherwise near the $+1/2$ defects, and we assumed the cell flow near the $-1/2$ defects was incompressible ($\langle \nabla \cdot \boldsymbol{v} \rangle = 0$). (d) Residual in velocity flux $\langle \Phi_{\boldsymbol{v}} \rangle$ for the $+1/2$ defect, which is the experimental value (red) minus the approximation (black) in (b).

Numerical scheme: To solve Eq. S23 with the constraint $\nabla \cdot \langle \boldsymbol{v} \rangle = j(\boldsymbol{r})$, we formally separate $\langle \boldsymbol{v} \rangle$ into $\langle \boldsymbol{v} \rangle = \langle \boldsymbol{u} \rangle + \langle \boldsymbol{w} \rangle$, where $\langle \boldsymbol{w} \rangle$ is an arbitrary velocity field that satisfies $\nabla \cdot \langle \boldsymbol{w} \rangle = j$. Specifically, we set $\langle \boldsymbol{w} \rangle = \boldsymbol{0}$ for $-1/2$ defects, and $\langle \boldsymbol{w} \rangle = w(r)\hat{\boldsymbol{r}}$ for $+1/2$ defects, where $w(r) = -j_0 r/2$ for $r \leqslant R_0$ and $w(r) = -j_0 R_0^2/(2r)$ for $r > R_0$. Next, the governing equation for $\langle \boldsymbol{u} \rangle$ becomes

$$\tilde{\xi}(\boldsymbol{I} - \epsilon \langle \boldsymbol{Q} \rangle) \cdot \langle \boldsymbol{u} \rangle = -\tilde{\xi}(\boldsymbol{I} - \epsilon \langle \boldsymbol{Q} \rangle) \cdot \langle \boldsymbol{w} \rangle - \nabla \cdot \langle \boldsymbol{Q} \rangle - \nabla \left\langle \tilde{P} \right\rangle, \text{ and } \nabla \cdot \langle \boldsymbol{u} \rangle = 0. \tag{S24}$$

Eq. S24 is solved using a semi-implicit Fourier spectral method as previously described [3, 4]. Briefly, we formally rewrite Eq. S24 as

$$a \langle \boldsymbol{u} \rangle - b\nabla^2 \langle \boldsymbol{u} \rangle = a \langle \boldsymbol{u} \rangle - b\nabla^2 \langle \boldsymbol{u} \rangle - \tilde{\xi}(\boldsymbol{I} - \epsilon \langle \boldsymbol{Q} \rangle) \cdot (\langle \boldsymbol{u} \rangle + \langle \boldsymbol{w} \rangle) - \nabla \cdot \langle \boldsymbol{Q} \rangle - \nabla \left\langle \tilde{P} \right\rangle \equiv -\nabla \left\langle \tilde{P} \right\rangle + \boldsymbol{F}(\langle \boldsymbol{u} \rangle), \tag{S25}$$

where a and b are introduced to stabilize the numerical scheme, and we have grouped all the non-pressure terms into $\boldsymbol{F}(\langle \boldsymbol{u} \rangle) = a \langle \boldsymbol{u} \rangle - b\nabla^2 \langle \boldsymbol{u} \rangle - \tilde{\xi}(\boldsymbol{I} - \epsilon \langle \boldsymbol{Q} \rangle) \cdot (\langle \boldsymbol{u} \rangle + \langle \boldsymbol{w} \rangle) - \nabla \cdot \langle \boldsymbol{Q} \rangle$. Taking the divergence of Eq. S25 and using the incompressibility condition $\nabla \cdot \langle \boldsymbol{u} \rangle = 0$, we obtain

$$-\nabla^2 \left\langle \tilde{P} \right\rangle + \nabla \cdot \boldsymbol{F} = 0. \tag{S26}$$

We denote by $[f]_{\boldsymbol{k}} = \int f(\boldsymbol{x}) e^{-i\boldsymbol{k}\cdot\boldsymbol{x}} d^2\boldsymbol{x}$ the Fourier transform of an arbitrary function $f(\boldsymbol{x})$. The Fourier transform of Eq. S26 leads to $|\boldsymbol{k}|^2[\left\langle \tilde{P} \right\rangle]_{\boldsymbol{k}} + i\boldsymbol{k} \cdot [\boldsymbol{F}]_{\boldsymbol{k}} = 0$. Introducing this relation into the Fourier transform of Eq. S25, we obtain that $(a + b|\boldsymbol{k}|^2)[\langle \boldsymbol{u} \rangle]_{\boldsymbol{k}} = (\boldsymbol{I} - \frac{\boldsymbol{kk}}{|\boldsymbol{k}|^2})[\boldsymbol{F}(\langle \boldsymbol{u} \rangle)]_{\boldsymbol{k}}$. Thus, $\langle \boldsymbol{u} \rangle$ can be solved using the following recursion relation

$$[\langle \boldsymbol{u} \rangle^{(m+1)}]_{\boldsymbol{k}} = \frac{1}{a + b|\boldsymbol{k}|^2}(\boldsymbol{I} - \frac{\boldsymbol{kk}}{|\boldsymbol{k}|^2})[\boldsymbol{F}(\langle \boldsymbol{u} \rangle^{(m)})]_{\boldsymbol{k}}, \tag{S27}$$

where the superscript $^{(m)}$ denotes the expression evaluated at the mth iteration step. The iteration ends when $\max |\, \langle \boldsymbol{u} \rangle^{(m+1)} - \langle \boldsymbol{u} \rangle^{(m)} \,| < 10^{-3} \max \left[\, \langle \boldsymbol{u} \rangle^{(m+1)} \,\right]$. We set $a = b = 3\tilde{\xi}$, and implemented the above numerical scheme in MATLAB.

Fitting to experimental data: We follow previous work [1] and use $\langle \boldsymbol{Q} \rangle = \Phi(r/\ell) \begin{pmatrix} \cos(2q\phi) & \sin(2q\phi) \\ \sin(2q\phi) & -\cos(2q\phi) \end{pmatrix}$ in Eq. S23 to solve for $\langle \boldsymbol{v} \rangle$. We treat ℓ, ξ_0,ϵ, and ζ_c as fitting parameters. We assume that $\pm 1/2$ defects have the same ℓ, ξ_0, and ϵ, but can have different ζ_c. The values of these fitting parameters are determined by minimizing the root-mean-square deviation between the model and the experiments, which yields $\ell = 2.0$ μm, $\epsilon = 0.35$, $\xi_0 = 6.5$ Pa $\cdot$ min/μm, and $\zeta_c = 3.8$ Pa $\cdot$ μm for $+1/2$ defects and $\zeta_c = 4.8$ Pa $\cdot$ μm for $-1/2$ defects.

III. BACTERIAL STRAINS

Wild type (WT) *M. xanthus* cells have two types of motility: social (S) motility and adventurous (A) motility. The former is driven by the type IV pili [5] and the latter by the gliding motors [6]. A strain with both S-motility and A-motility is called A^+S^+. Besides WT, we used various genetically modified strains in the experiments for different purposes.

To simplify the intercellular and cell-substrate interactions, we used a Δ*pilA* mutant, which does not make any type IV pilus and thus has no S-motility (A^+S^-). Pili normally extrude from the leading pole of the cell and can extend longer than a cell length. They can attach to the substrate and to other cells, and the cell can generate a pulling force by retracting the pili. Such an interaction could act as a contractile active force that pulls the cells closer to each other in both the tangential and transverse directions. However, we do not know the number of active pili per cell, their pulling force, or their pulling rate under our experiments' conditions, which makes it difficult to incorporate these factors into our current model. The Δ*pilA* mutant has simpler cell-cell and cell-substrate interactions compared to the WT – the active force is only generated via direct contact and each cell can only interact with the substrate or its neighbors. In Fig. S12, we compare the experimentally measured forces generated by thin colonies of WT and Δ*pilA* cells to demonstrate the effects of pili. As expected, the traction and velocity perpendicular to the long axes of the WT cells have a poorer correlation than the Δ*pilA* cells, which implies that the WT cells apply extra sideways active forces on their neighbors. Nevertheless, overall, the behavior of A^+S^- cells in a thin layer is very similar to WT (see Fig. S13 and Fig. S14), so we used an A^+S^- strain as the reversing cells to obtain the data in the main text.

To test the effect of cell reversal in Fig. 4 (main text), as the non-reversing cells we used a Δ*frzE* mutant (A^+S^+), which reverses the direction of motion much less frequently compared to WT and Δ*pilA* cells in a nutrient-rich environment. Figure S13 shows a comparison of the WT, Δ*pilA*, and Δ*frzE* strains, demonstrating that the increased polarity, layering, and stress we see in the non-reversing mutant is not a function of the presence of pilus-driven S-motility in the Δ*frzE* strain. To measure the cell polarity, we used a *mglB*::*mVenus* strain with fluorescent labels on the MglB protein, which localizes to the lagging pole of the cell. These *mglB*::*mVenus* cells are A^+S^+ and their behaviors are very close to WT. The strains and their properties are summed up in Table S2.

Strain	Motility	With pilus?	Reversal?	Fluorescent label	Reference
WT (DK1622)	A^+S^+	Yes	Yes	No	[1]
Δ*pilA*	A^+S^-	No	Yes	No	[7]
Δ*frzE*	A^+S^+	Yes	No	No	[8]
mglB::*mVenus*	A^+S^+	Yes	Yes	MglB	[9]

TABLE S2: *M. xanthus* strains used in the experiments.

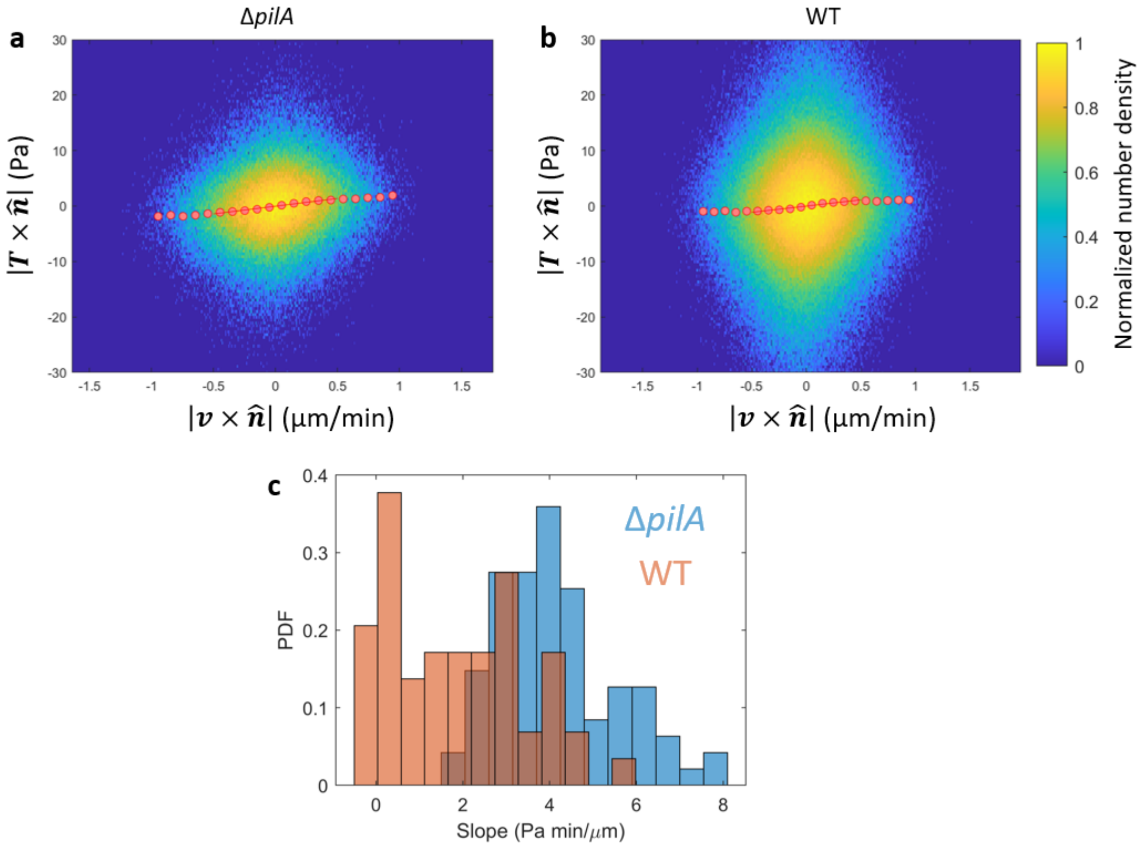

Fig. S12: Effects of type-IV pili on the instantaneous traction force generated by the bacteria. (a,b) Relationship between the components of traction force $\boldsymbol{T}$ and cell velocity $\boldsymbol{v}$ perpendicular to the local director $\hat{\boldsymbol{n}}$. These perpendicular components $|\boldsymbol{T} \times \hat{\boldsymbol{n}}|$ and $|\boldsymbol{v} \times \hat{\boldsymbol{n}}|$ are measured point by point in the videos. The color map shows the normalized number density of a location with certain $|\boldsymbol{T} \times \hat{\boldsymbol{n}}|$ and $|\boldsymbol{v} \times \hat{\boldsymbol{n}}|$. (a) is obtained with the $\Delta pilA$ strain and (b) is with the WT. We fitted the middle sections of the mean data (red circles) in (a) and (b), and did this for all the videos that we took, and obtained the slopes shown in (c). The ratio between $|\boldsymbol{T} \times \hat{\boldsymbol{n}}|$ and $|\boldsymbol{v} \times \hat{\boldsymbol{n}}|$ provides a viscous-like friction coefficient. The results obtained with the $\Delta pilA$ cells clearly deviate from zero, which means that the transverse components of velocity and traction are correlated. This is consistent with transverse tractions being due to just viscous friction, with no contribution from active forces. In the absence of pili, active forces arise from cell gliding, and they are therefore along the cell body axis, and hence along the director $\hat{\boldsymbol{n}}$. However, for the WT cells, the correlation between transverse traction and velocity becomes significantly weaker because now a cell can potentially pull on its lateral neighbors using type-IV pili and thus generate active forces perpendicular to its long axis. This effect covers up the relationship between $|\boldsymbol{T} \times \hat{\boldsymbol{n}}|$ and $|\boldsymbol{v} \times \hat{\boldsymbol{n}}|$ given by just cell-substrate friction. To avoid this complication, we used the $\Delta pilA$ strain instead of the WT as the model organism in this paper. Nevertheless, we measured velocity and traction fields around defects in WT colonies, as shown in Fig. S14, which show no qualitative difference from those obtained with the $\Delta pilA$ mutant (Fig. 1 and Fig. 3 in the main text and Fig. S1).

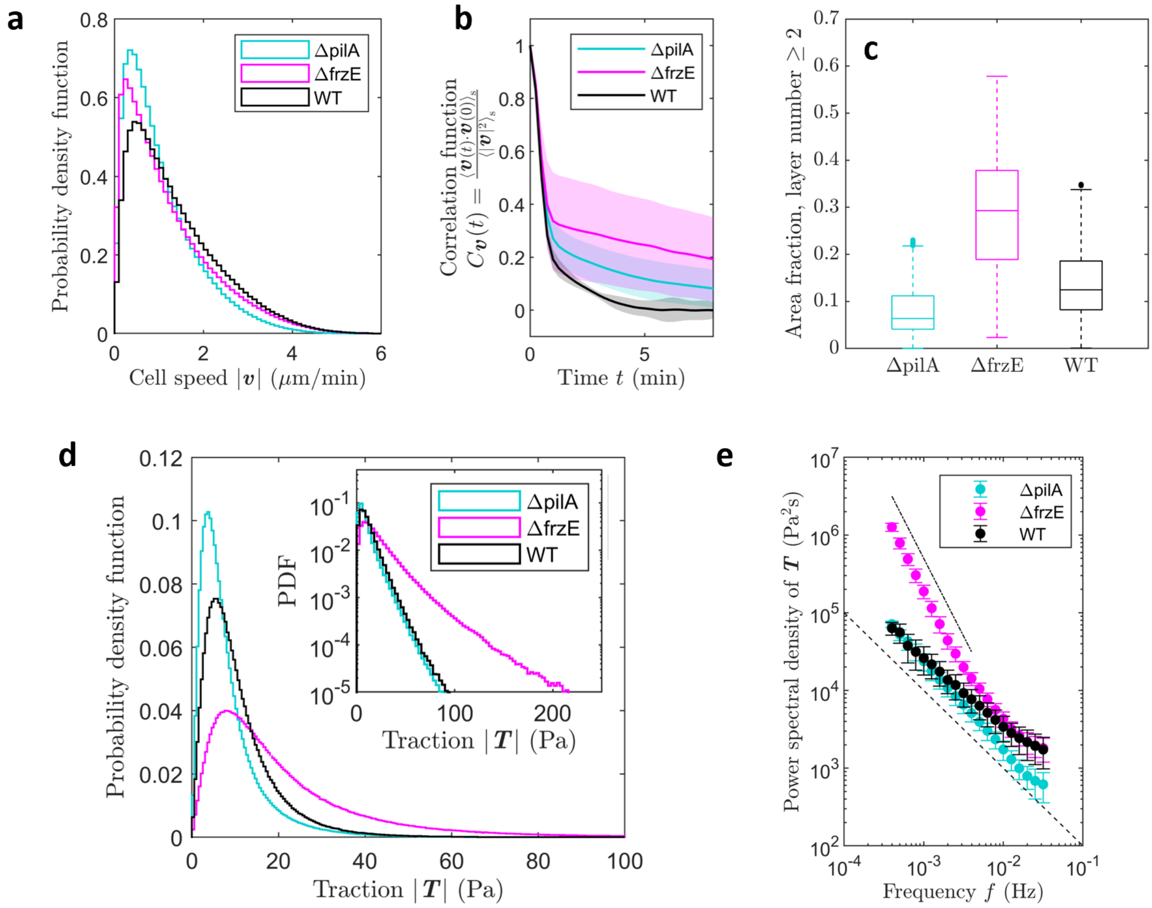

Fig. S13: Compare three different strains: WT, Δ*pilA*, and Δ*frzE*. The WT and Δ*pilA* cells reverse, but the Δ*frzE* cells do not. The WT and Δ*frzE* cells have type-IV pili, but the Δ*pilA* cells do not. In all the experiments, the cells stayed in a nutrient rich environment. (a) Probability distribution functions of cell speed $|\boldsymbol{v}|$ in thin colonies. The difference between these three strains was minor. (b) Temporal correlation functions of cell velocity $C_v(t) = \frac{\langle \boldsymbol{v}(t)\cdot\boldsymbol{v}(0)\rangle_{\mathrm{s}}}{\langle|\boldsymbol{v}|^2\rangle_{\mathrm{s}}}$, where $\langle\ \rangle_{\mathrm{s}}$ represents averaging across different areas. The solid curves show the means and the shaded areas show the standard deviations. Similar to Fig. S6, we fit each $C_{\boldsymbol{v}}(t)$ to a stretched exponential function $C(t) = \exp\left[-(t/\tau)^{\beta}\right]$. The WT cells had the minimum average correlation time $\overline{\tau}$, then it was the Δ*pilA* cells, and they both had shorter $\overline{\tau}$ than the Δ*frzE* cells. (c) Areal ratio of regions where the cell colony was thicker than a monolayer ($n_{\mathrm{L}} \geq 2$) in the whole field of view of the videos. The Δ*frzE* cells created more areas where the cells aggregated into multiple layers compared to the WT and Δ*pilA* cells. (d) Probability distribution functions of traction magnitudes $|\boldsymbol{T}|$ for the three strains. The inset shows the same data in log-linear scales. The difference between the WT and Δ*pilA* cells was marginal, but they both had significantly narrower distributions than the Δ*frzE* strain. (e) Power spectral densities (PSD) of traction $\boldsymbol{T}$ for all three strains. The PSD of WT approached that of Δ*pilA* in the low-frequency regime, while it approached that of Δ*frzE* in the high-frequency regime. This suggests that the cell reversal controls the low-frequency component of the mechanical stress in the colonies and the pili only affect the high-frequency regime.

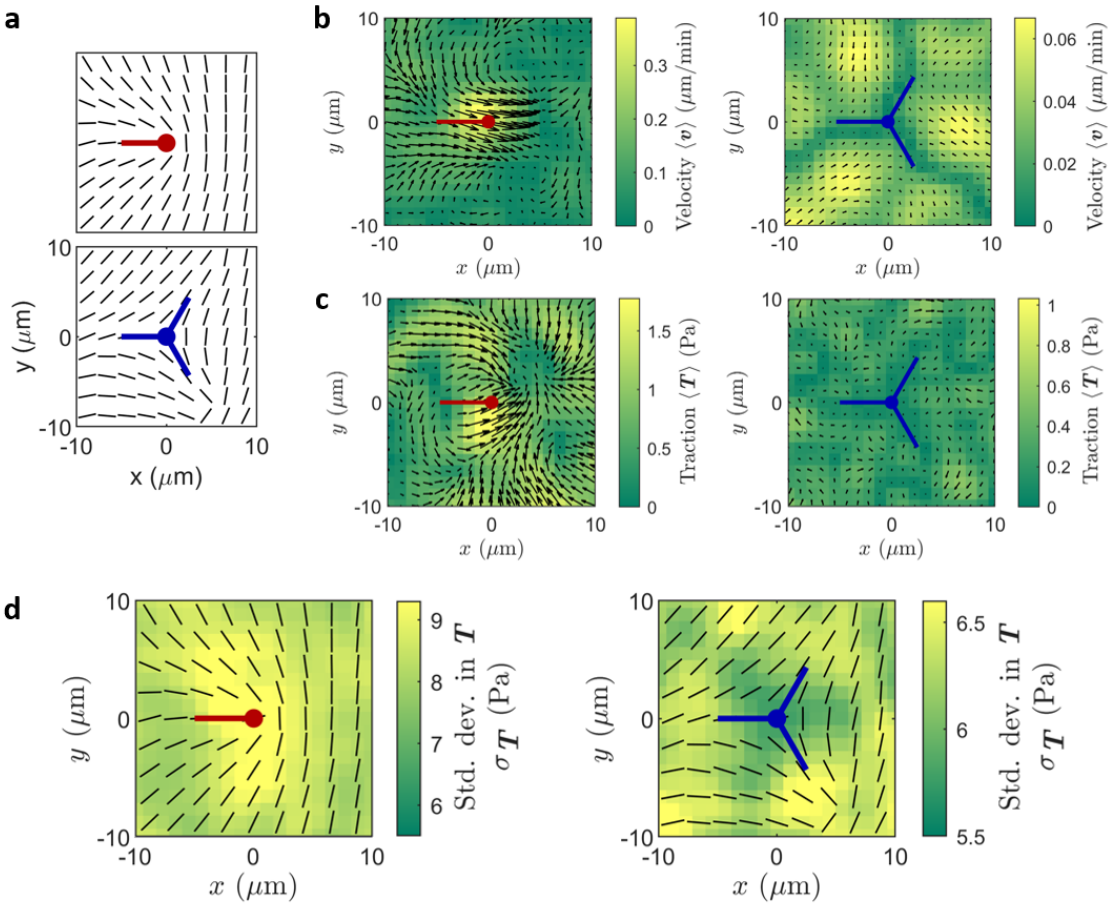

Fig. S14: Director, velocity, traction, and traction fluctuation fields around ±1/2 defects in thin colonies of WT cells (A^+S^+ strain with reversal). (a) Experimentally measured mean director field $\langle \hat{\boldsymbol{n}} \rangle$ around ±1/2 defects. (b) Experimentally measured mean velocity field $\langle \boldsymbol{v} \rangle$ near ±1/2 defects. The black arrows show its magnitude and direction and the color map shows the speed $|\langle \boldsymbol{v} \rangle|$. (c) Experimentally measured mean traction field $\langle \boldsymbol{T} \rangle$ around ±1/2 defects. The color maps show their magnitudes, and the arrows indicate their magnitude and direction. (d) Experimentally measured standard deviation of traction, $\sigma_{\boldsymbol{T}}$, around ±1/2 defects. The black lines show the director field $\langle \hat{\boldsymbol{n}} \rangle$.

IV. MEASURING DIRECTOR AND VELOCITY FIELDS FROM IMAGES OF CELLS

A. Processing bright field images of the cells

We used the bright field images to obtain the director field and cell flow velocity. We started with some basic processing steps, including removing the slowly varying background and adjusting the contrast. We also removed the slow global drift measured using the fluorescence images as described in Section VI A. An example of the processed bright field images is shown in Fig. S15a.

B. Detecting holes in cell layers

We used the local maximum difference in brightness to separate holes (bare gel surface) from the cell layer. In a region with cells, this difference is more substantial than for the bare gel surface (holes in the cell layer), where the brightness is more uniform. For each pixel, we calculated the maximum and minimum brightness, $I_{\text{local, max}}$ and $I_{\text{local, min}}$, respectively, within a 10×10 pixel box centered at this pixel. Then we rescaled the brightness difference using

$$I_{\text{diff}} = 1 - \text{normalize}(I_{\text{local, max}} - I_{\text{local, min}}), \tag{S28}$$

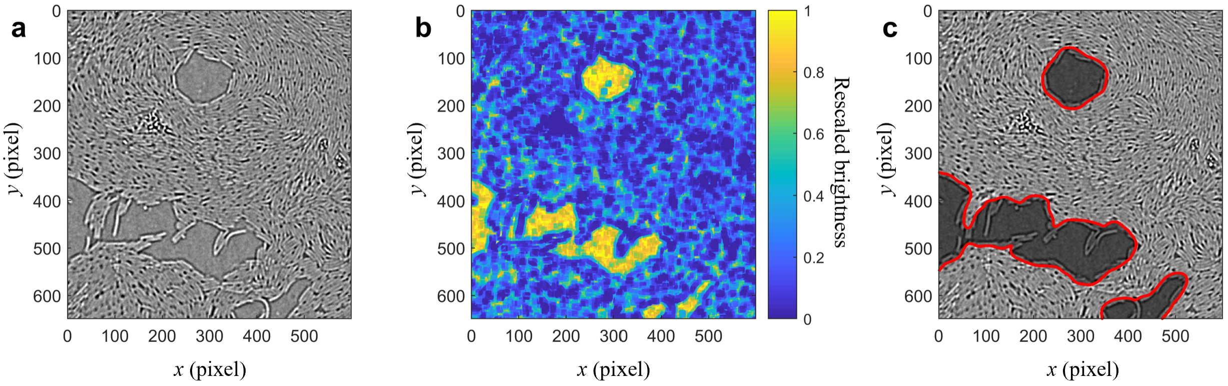

Fig. S15: Detecting holes in the bacterial colony. This example was imaged at 60× magnification, and the length scale is 0.11 μm per pixel. (a) Bright field images of the cells. (b) Rescaled brightness I_{diff} (Eq. S28). (c) Final result: the holes are highlighted with darker gray and red edges.

where "normalize" means adjusting the brightness of the image ($I_{\text{local, max}} - I_{\text{local, min}}$) so that it ranges between 0 and 1. The rescaled image I_{diff} enhances the difference between cell layers and holes as shown in Fig. S15b. We then set a threshold and binarized I_{diff} to distinguish holes from cell layers.

In this project, we care more about the regions where there is a densely packed cell layer and the $\pm 1/2$ topological defects in these regions. However, sometimes our algorithm recognized some features inside or on the edges of the holes as $\pm 1/2$ defects, and such defects were to be excluded from our analysis. As a result, we dilated the regions recognized as holes so that their boundaries slightly extended beyond the actual edges of the holes. This allowed us to only keep the $\pm 1/2$ defects located inside the cell layer. We also excluded the holes with an area smaller than 900 pixels (about 11 μm^2). Consequently, when the cell layer was slightly cracked open in a small region due to a fluctuation in cell concentration, it was still counted as a cell layer. Lastly, we smoothed the edges of the holes both in space and time using a Gaussian filter. The detected holes are labeled in Fig. S15c.

C. Nematic order

To characterize the nematic order within a cell layer, we first measured the cell orientation angle θ_{ij} following [1, 10] using the pre-processed bright field image. In an image, the brightness of the pixel in row i and column j is I_{ij}. We calculated the Hessian matrix H_{ij} pixel by pixel, where

$$H_{ij} = \begin{pmatrix} (\partial I_{ij}/\partial x)^2 & (\partial I_{ij}/\partial x)(\partial I_{ij}/\partial y) \\ (\partial I_{ij}/\partial x)(\partial I_{ij}/\partial y) & (\partial I_{ij}/\partial y)^2 \end{pmatrix}, \tag{S29}$$

and smoothed each element in the matrix by means of a Gaussian filter with standard deviation $\sigma = 10$ pixels (1.1 μm for 60× magnification). The eigenvector associated with the smallest eigenvalue of H_{ij} gives the local direction of the smallest brightness gradient, which was taken to represent the cell orientation. An exemplary director field is shown in Fig. S16b, based on the bright field image in Fig. S16a.

D. Detection and tracking of topological defects

From the measured θ_{ij}, we obtained the scalar order parameter S:

$$S_{ij} = \sqrt{\langle\cos(2\theta_{ij})\rangle_R^2 + \langle\sin(2\theta_{ij})\rangle_R^2}, \tag{S30}$$

where $\langle\,\rangle_R$ represents averaging within a disk of radius $R = 5$ pixels (0.55 μm for 60× magnification) centerd at the pixel (i, j). As shown in Fig. S16c, S_{ij} vanishes near the cores of defects and is approximately 1 everywhere else. For each point identified as a potential defect core, we calculated the topological charge q by applying its definition:

$$q = \frac{1}{2\pi}\oint_{\mathcal{C}} \mathrm{d}\theta, \tag{S31}$$

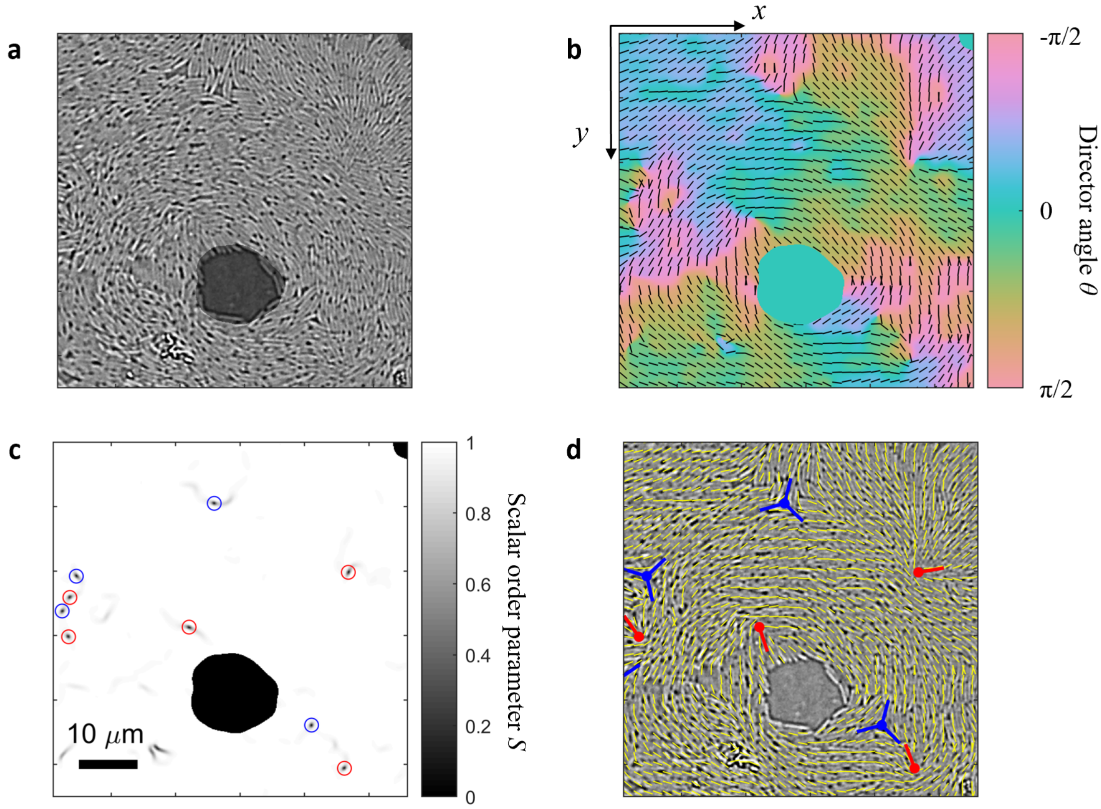

Fig. S16: Measuring director field θ and detecting $\pm 1/2$ defects. (a) Bright field image of the cells, with the holes shaded in a darker color. (b) Director field θ showing the orientation of the cells labeled by both the color map and the short black lines. The director is horizontal when $\theta = 0$ and θ increases when the line rotates clockwise. (c) Scalar order parameter S. We label the regions recognized as defects with circles: red circles represent $+1/2$ defects and blue circles represent $-1/2$ defects. The holes are excluded. (d) Directors (yellow lines) overlaid on the bright field image of the cells. The locations and orientations of the topological defects are labeled using the same color as in (c). The scale bar in (c) is 10 μm and applies to all panels.

where $\mathcal{C}$ is a circular circuit of radius $R_c = 6$ pixels (0.66 μm at 60×) around the defect core. We ignored the candidate points with $q = 0$. For each defect, we identified its axes of symmetry using the method in Refs. [1, 11]. Specifically, the axis of a $+1/2$ defect is given by $\hat{p}_\alpha = \partial_\beta Q_{\alpha\beta}/|\partial_\beta Q_{\alpha\beta}|$, where Q is the nematic order parameter tensor with components

$$Q_{\alpha\beta} = S\left[2\hat{n}_\alpha \hat{n}_\beta - \delta_{\alpha\beta}\right], \tag{S32}$$

where $\hat{\mathbf{n}} = (\cos\theta,\ \sin\theta)$. Similarly, for $-1/2$ defects, we first defined the opposite nematic angle $\theta' = -\theta$, obtained the corresponding Q' tensor, and then calculated $\hat{p}'_\alpha = \partial_\beta Q'_{\alpha\beta}/|\partial_\beta Q'_{\alpha\beta}|$. Finally, defining $\hat{\mathbf{p}}' = (\sin\psi',\ \cos\psi')$, one symmetry axis of a $-1/2$ defect is given by the angle $\psi = -\psi'/3$. The other two symmetry axes of a $-1/2$ defect were then found by its three-fold symmetry.

We tracked the defects' motion using Blair and Dufresne's particle tracking velocimetry (PTV) code [12]. We ignored defects that exist for shorter than eight frames (2 minutes). We also ignored defects in holes, and those within 50 pixels of the edges of the images. Figure S16d shows the director field obtained from the bright field image, and the locations and orientations of the detected $+1/2$ (red) and $-1/2$ (blue) defects. One can see that some of the singular points circled out in Fig. S16c are not shown in Fig. S16d, because they did not last long enough and thus were excluded.

E. Measuring velocity and cell flows

We used the method of optical flow to measure cell velocity. After processing the original bright field images of the cells, we used the MATLAB (R2019b) function opticalFlowFarneback() to obtain the velocity field. Figure S17a shows an exemplary image of the cells with the measured velocity vectors overlaid. The parameters for the optical flow were calibrated using images of a *frzS*::*GFP* strain. The fluorescently labeled FrzS proteins generated bright spots at both poles of the cells, which allowed us to measure the same cell flow fields with

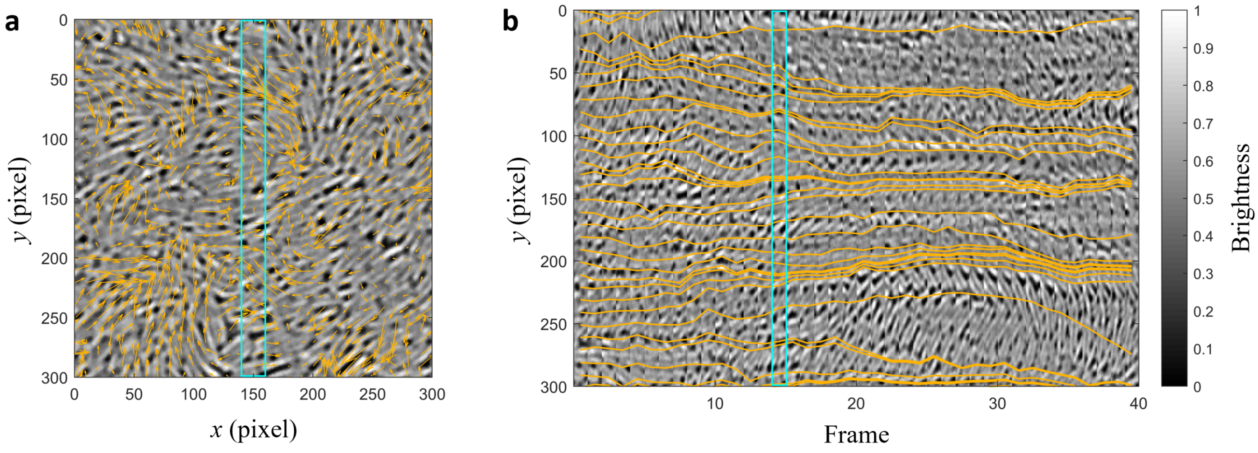

Fig. S17: Using optical flow to measure the velocity of cell flow. (a) Processed bright field image of the cells with the orange velocity vectors overlaid. (b) Kymograph obtained by stitching together the same rectangular region (surrounded by lines in cyan in (a)) in the bright field image series in chronological sequence. This selected region is 300 pixels tall and 10 pixels wide shown by the cyan rectangle in (a) and (b). The cyan rectangular regions in (a) and (b) are identical. The overlaid orange curves are the time integral of the y velocity measured at different y positions.

two different methods: using PIV for the fluorescent images and optical flow for the bright field images. The two methods provided the same measurements in most regions. In the end, we calculated the displacement by integrating the velocity over time and overlaying the displacement curves on top of the kymograph made from the bright field images (Fig. S17b). These orange displacement curves nicely follow the up-and-down movement of the speckles in the kymograph.

V. MEASURING CELL POLARITY

A. Single-cell polarity vs. velocity

The *mglB*::*mVenus* strain that we used was generated and characterized by Szadkowski *et al.* [9]. We tested the strain using samples with sparse cells on the surface, and indeed, the direction of motion of a single cell was highly correlated with the position of the MglB protein: MglB localized to the lagging pole of the cell (Fig. S18a).

B. Data processing for cell layers

Combining the fluorescence images of the fluorescent labels and the bright-field images of the cells, we measured cell polarity $\boldsymbol{p}$. One way to achieve this is to perform cell segmentation and determine the location of MglB within each cell. However, this was difficult with our bright-field images, so we used another method that measured the local polarity without cell segmentation. First, we processed the fluorescence images with a band-pass filter to denoise and enhance their contrast, then we located the centers of the fluorescent MglB labels (Fig. S18b). Next, we measured the local directors at the locations of these centers, as shown by the yellow lines in Fig. S18c. As Fig. S18d shows, since MglB always appeared close to a cell pole, for each MglB focus (green dot), we compared the brightness in the bright-field image along the corresponding yellow line. Because the gap between adjacent cells had lower brightness, the brighter side indicated the inside of the cell, and the darker side indicated the outside. Since MglB localized to the lagging pole of the cell, we obtained the polarity vector $\boldsymbol{p}$ pointing along the direction of the yellow line toward the brighter side.

As shown in Fig. S18e-g, in each frame, we obtained the cell polarity (green arrows) and velocity (orange arrows, see SI Sec. IV) simultaneously. When comparing cell polarity and velocity, we focused on two special cases: ordered regions where the cells were approximately parallel to each other (Fig. S18e and f) and regions around +1/2 defects (Fig. S18g). In ordered regions, we observed both polar flows and nematic flows, meaning the cells moving in the same direction (Fig. S18e) and opposite directions (Fig. S18f), respectively. To quantify the local polar order, we reoriented the cells to become horizontal, as shown in Fig. S19a. We first calculated the mean director $\langle\hat{\boldsymbol{n}}\rangle_{\mathrm{s}}$, and then rotated the image such that $\langle\hat{\boldsymbol{n}}\rangle_{\mathrm{s}}$ became horizontal. Then we took the

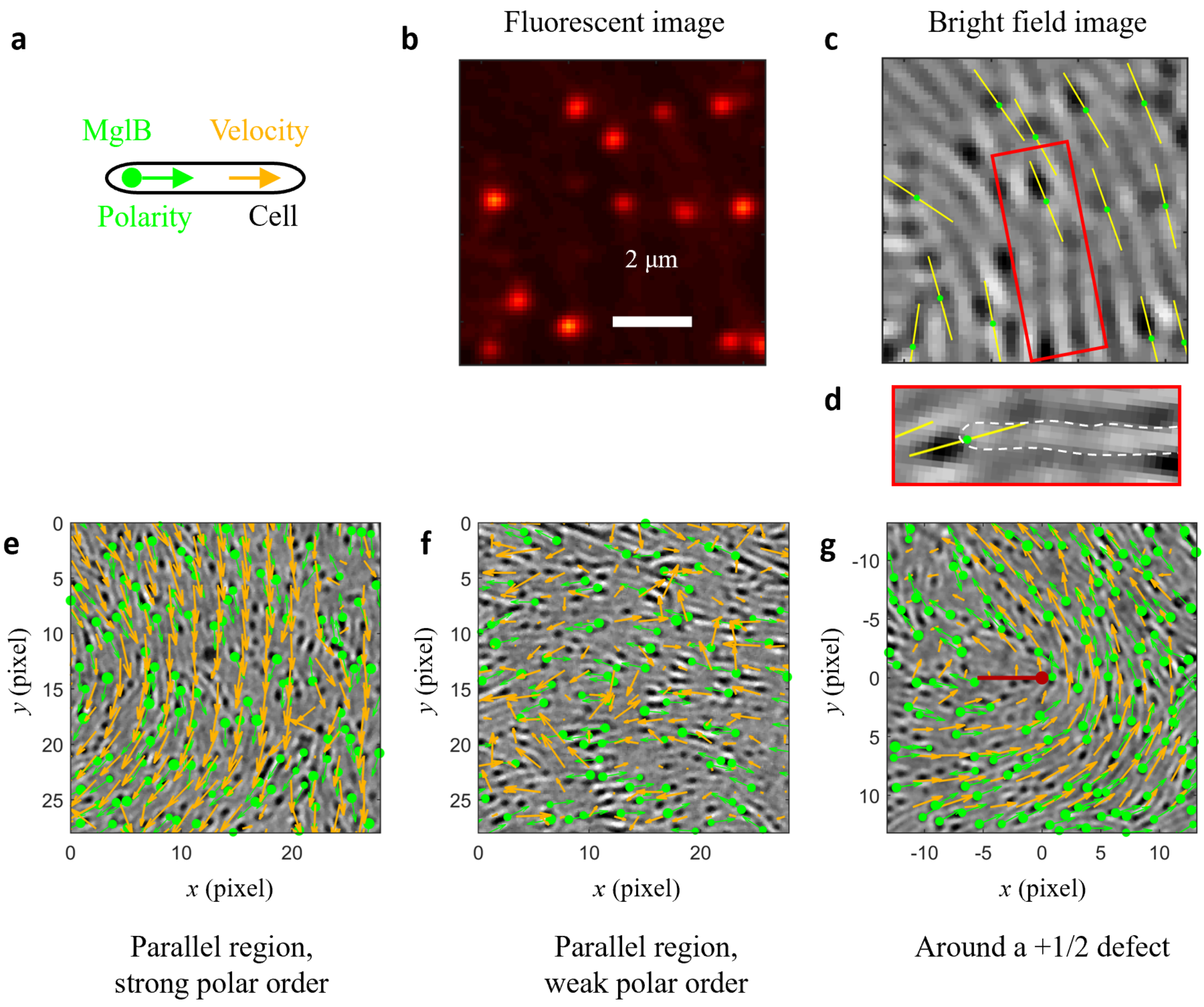

Fig. S18: Cell polarity measurement. (a) A schematic illustration of the relationship between cell velocity and the location of the fluorescently labeled MglB protein for a single cell. (b) Fluorescence image of the labeled MglB proteins. The scale bar represents 2 μm. (c) Bright-field image of the cells in (b) with the positions of the MglB labels (green dots) overlaid. The yellow lines show the local directors $\hat{\boldsymbol{n}}$. (d) Reoriented region inside the red box in (c) with one cell body outlined by the white dashed line. (e-g) Polarity (green) and velocity (orange) vectors overlaid on bright-field images. The green dots show the MglB labels and their sizes represent the sizes of the bright MglB spots in the corresponding laser images. The three types of flow: (e) ordered region with cells moving in the same direction; (f) ordered region with cells moving in opposite directions; (g) near a +1/2 defect.

horizontal components of the reoriented polarity p_{h} and velocity v_{h} inside a $12 \times 12\ \mu\mathrm{m}^2$ square boxes (two cell lengths) and obtained $p_n = \langle p_{\mathrm{h}} \rangle_{\mathrm{s}}$ and $v_n = \langle v_{\mathrm{h}} \rangle_{\mathrm{s}}$, where $\langle\ \rangle_{\mathrm{s}}$ denote spatial average inside the box. Note that $\langle \hat{\boldsymbol{n}} \rangle_{\mathrm{s}}$ has two identical ends, so in the aligned regions, even though we have a cell orientation, we cannot define which direction is positive or negative. As a result, we combined the data (p_n, v_n) and $(-p_n, -v_n)$ in Fig. 1f of the main text and in Fig. S19a. In an area around a $+1/2$ defect, we reoriented the cells so that the "comet head" was on the right-hand side and the "comet tail" on the left, and the tail became horizontal, as shown in Fig. S19b. We calculated $p_n = \langle p_{\mathrm{h}} \rangle$ and $v_n = \langle v_{\mathrm{h}} \rangle$ in the same way, but now the square box is explicitly chosen in the comet tail region. Since the director field of the $+1/2$ defect breaks the spatial symmetry, now we can define the positive direction as pointing to the right, as indicated by the arrow in Fig. S19b.

VI. MEASURING TRACTION FROM FLUORESCENCE IMAGES

The fluorescence images captured the fluorescent particles close to the surface of the gel. We used particle imaging velocimetry (PIV) to track their motion and obtained the displacement field of the substrate in the x-y plane, which is parallel to its surface. Then we used the displacement field to reconstruct the traction field. In Section VII, we discuss measuring the deformation of the substrate in the direction normal to its surface (the

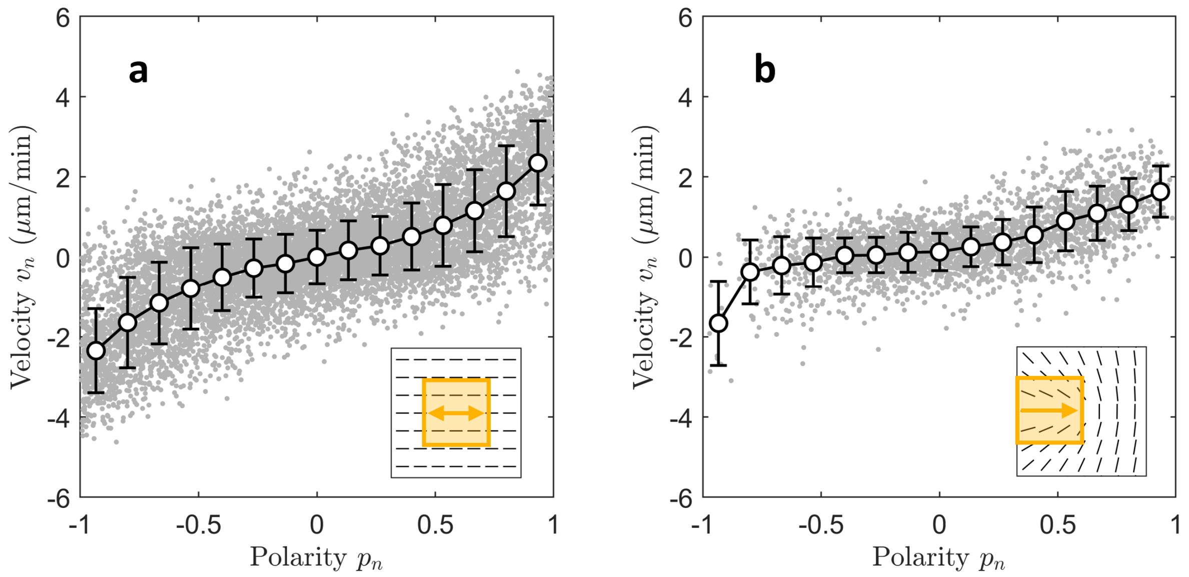

Fig. S19: Relationship between polarity and velocity in ordered regions and near $+1/2$ defects obtained with the polarity assay. (a) Relationship between the local mean velocity v_n and polarity p_n in ordered regions, which were calculated in 12×12 μm^2 boxes (orange square in the inset). First, we reoriented the area so that the cells aligned horizontally. Then we used the horizontal component of velocity $v_{\rm h}$ and polarity $p_{\rm h}$ to obtain $v_n = \langle v_{\rm h} \rangle_{\rm s}$ and $p_n = \langle p_{\rm h} \rangle_{\rm s}$, where the mean was calculated within the square. Each gray point in the plot was obtained with one of such squares. Note that the directors $\langle \hat{\boldsymbol{n}} \rangle_{\rm s}$ and $-\langle \hat{\boldsymbol{n}} \rangle_{\rm s}$ are equivalent, so here we show both (p_n, v_n) and $(-p_n, -v_n)$. The black circles and curve show the mean v_n at different p_n, and the error bars show the corresponding standard deviation. (b) Relationship between the local mean velocity v_n and polarity p_n in the tail region of $+1/2$ defects (orange square in the inset, also 12×12 μm^2 in area). The symbols are the same as in (a). Similar to the aligned regions, here we reoriented the areas so that they look like the inset, and then we calculated $v_n = \langle v_{\rm h} \rangle_{\rm s}$ and $p_n = \langle p_{\rm h} \rangle_{\rm s}$. Different from the ordered case, here the defect breaks the spatial symmetry, so we defined a positive direction: v_n and p_n are positive when pointing to the right and negative when pointing to the left based on the orientation of the inset. Besides the orange square, we tried a triangular area in the tail region with the same area when performing these calculations, and the results showed no qualitative difference.

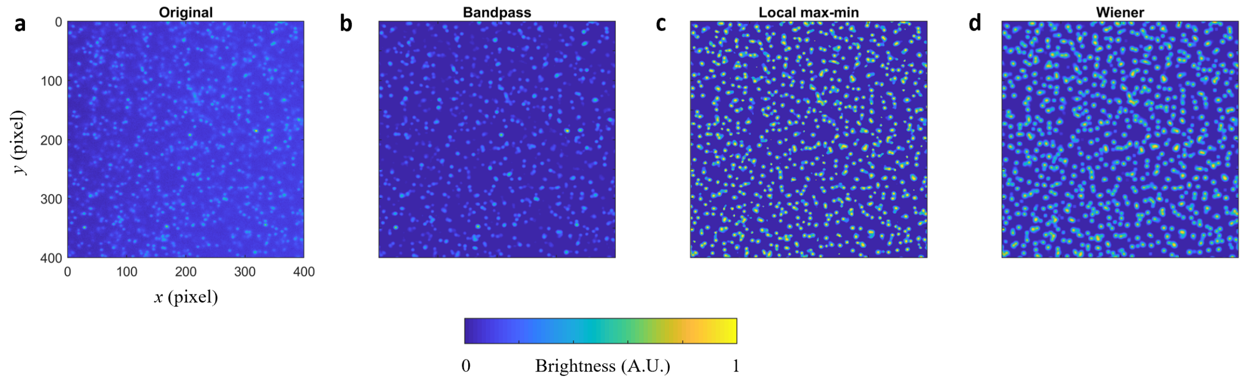

Fig. S20: Images of the 110 nm fluorescent particles at 60× magnification before (a) and after each step of processing: bandpass filter (b), local max-min filter (c), and Wiener filter (d). The length scale is 0.11 μm/pixel.

z direction).

A. Pre-processing fluorescence images

Before running the PIV algorithm, we processed the images using a band pass filter, a local min-max filter, and a Wiener filter in this order. For the bandpass filter, we used the bpass() function in the particle tracking velocimetry (PTV) code written by Blair and Dufresne [12]. The window sizes of the filters were chosen to be larger than the size of individual particles but smaller than the interrogation box in PIV [13]. Exemplary

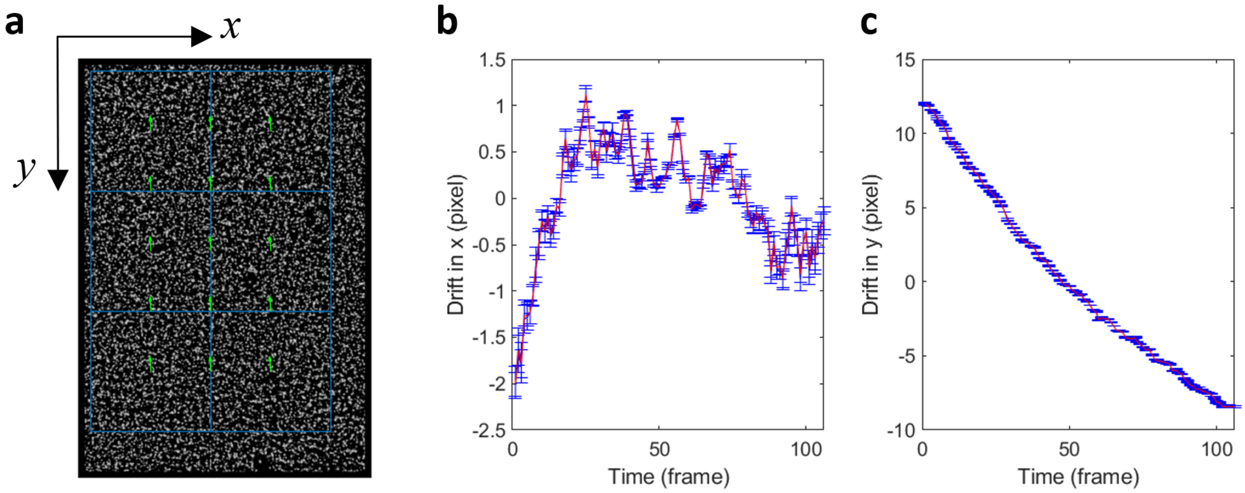

Fig. S21: Global drift in x and y directions of an exemplary video. (a) Displacement of frame 11 with respect to the reference frame. The reference frame was frame 53 in this case. The blue lines show the interrogation boxes and the green arrows show the displacement in every box. (b) Average displacement in the x direction. The red line shows the relative displacement averaged across all the interrogation boxes, and the blue error bars show the standard deviation. (c) Average displacement in the y direction. The labels are identical to (b).

images in Fig. S20 show the original and processed fluorescent images after every step for 110 nm beads at 60× magnification.

Even though we sealed the whole sample, the hydrogel still dehydrated slowly from time to time. As a result, in some videos, the substrate had a mild drift, where both the cells and the tracer particles moved together slowly in the field of view. An example of this global drift is shown in Fig. S21. Within 100 frames (about 25 min), the sample drifted approximately 20 pixels (about 1.5 μm) in the y direction, while remaining relatively steady in the x direction. We removed such global drifts by performing a low-resolution PIV with 400 pixels × 400 pixels interrogation boxes, calculating the mean displacement in all the boxes in each image, and cropping the images using a moving window that follows this drift.

B. Measuring displacement at the substrate surface

The displacement of the substrate parallel to its surface was calculated using a custom PIV algorithm. In the PIV analysis, we used an interrogation box of 20×20 pixels with an overlap (oversampling) of 50% (10 pixels). Fig. S22a and b present exemplary kymographs showing the motion of the speckles in a column of interrogation boxes in the x and y directions, respectively. The corresponding PIV results are overlaid to show a reasonably good agreement. To further reduce the noise in the measured displacement, we used multiple reference images while performing PIV. The gap between adjacent reference images was 20 frames. For example, for a video with 117 images, we made frames 1, 21, 41, ... 101 the reference images. Each reference image i was used to calculate the displacement field in the frames $i - 40$ to $i + 40$. Consequently, the displacement field in each frame of the video was calculated independently multiple times with different reference images. Then we combined the results obtained from two adjacent reference images by matching the mean displacement within the overlapped frames. The mean displacement in each frame was then calculated after removing the outliers. Lastly, we shifted the displacement of the first frame to zero everywhere, so the displacement calculated was all with respect to the first frame of the video. Fig. S22c shows how the x and y displacements obtained with different reference images are aligned and stitched together.

C. Traction reconstruction

Based on the x and y displacement fields obtained in the previous step, we reconstructed the traction map following the Green's function based method described in [14–16]. When a point force $\boldsymbol{F} = F_x\hat{x} + F_y\hat{y} + F_z\hat{z}$ ($\hat{x}$, $\hat{y}$, and $\hat{z}$ are unit vectors) is applied on the surface of an elastic medium occupying a half-space, the resulting

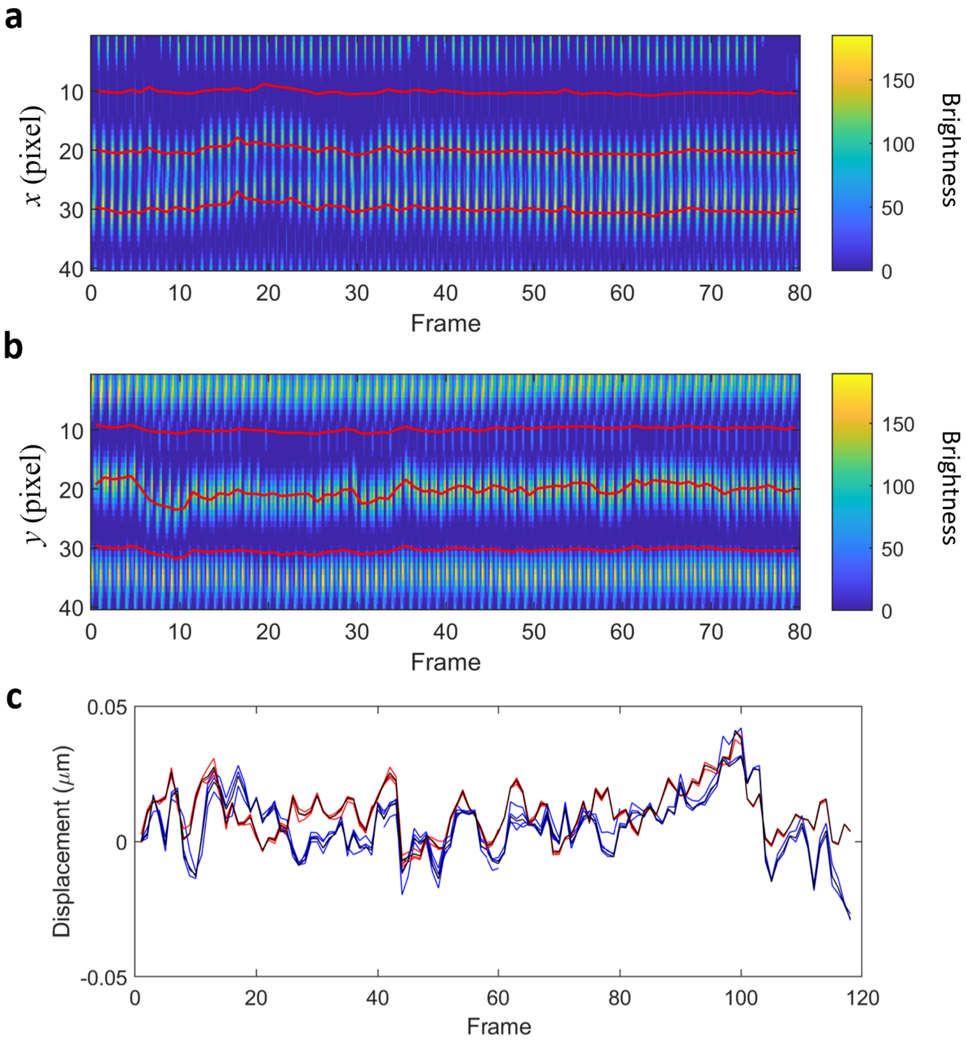

Fig. S22: Tracking the motion of particles. (a, b) Kymographs of speckle motion in the x (a) and y (b) directions. The local displacement calculated with the PIV algorithm is shown by the red curves. For each frame, we show an area of two interrogation boxes tall (40 pixels) and one box wide (20 pixels). The color map represents the brightness of the images. (c) An example of x (blue) and y (red) displacements calculated with different reference frames and stitched together. The black curves show the average displacements.

displacement field $\boldsymbol{u} = u_x\hat{x} + u_y\hat{y} + u_z\hat{z}$ is

$$u_x = \frac{3}{4\pi E}\left\{\frac{xz}{r^3}F_z + \frac{1}{r}F_x + \frac{x}{r^3}(xF_x + yF_y)\right\}, \tag{S33a}$$

$$u_y = \frac{3}{4\pi E}\left\{\frac{yz}{r^3}F_z + \frac{1}{r}F_y + \frac{y}{r^3}(xF_x + yF_y)\right\}, \tag{S33b}$$

$$u_z = \frac{3}{4\pi E}\left\{\left[\frac{1}{r} + \frac{z^2}{r^3}\right]F_z + \frac{z}{r^3}(xF_x + yF_y)\right\}. \tag{S33c}$$

where E is the Young's modulus, and $r = \sqrt{x^2 + y^2 + z^2}$ [17]. Since the hydrogel is nearly incompressible, we set the Poisson's ratio $\nu = 0.5$ in these equations. When we look at the surface of the gel where $z = 0$, the

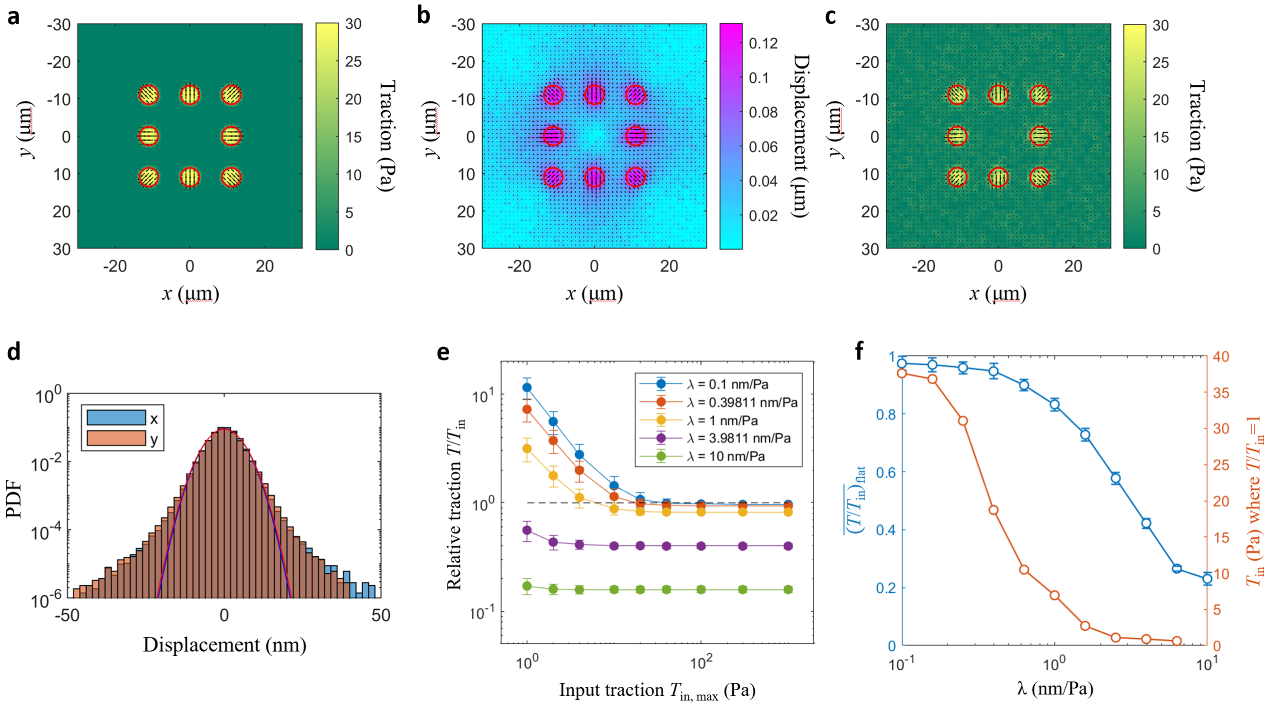

Fig. S23: Find regularization parameter λ using artificially generated traction map. (a) Input traction map. (b) Coarse-grained displacement field with noise. (c) Reconstructed traction map with $\lambda = 1$ nm/Pa. (d) Distribution of x (blue) and y (orange) displacement when there was no cell on the surface of the gel at 60× magnification. (e) Ratio between the reconstructed and the input traction magnitudes T/T_{in} in the regions where $T_{\text{in}} > 15$ Pa (50% peak value) as functions of T_{in} when reconstructed with different λ. (f) We obtained where $T/T_{\text{in}} = 1$ in (e). On the right-hand side of these intersections, the curves are approximately flat. Here we show the mean and standard deviation of the ratio T/T_{in} in the flat regime (blue), and the input T_{in} at which $T/T_{\text{in}} = 1$ (orange).

transverse direction (x and y) and the normal direction (z) are independent.

$$u_x = \frac{3}{4\pi E}\left\{\frac{1}{r}F_x + \frac{x}{r^3}(xF_x + yF_y)\right\}, \tag{S34a}$$

$$u_y = \frac{3}{4\pi E}\left\{\frac{1}{r}F_y + \frac{y}{r^3}(xF_x + yF_y)\right\}, \tag{S34b}$$

$$u_z = \frac{3}{4\pi E}\frac{F_z}{r}. \tag{S34c}$$

In two dimensions, the relationship between the tangential traction $\boldsymbol{T}(x, y, t)$ (local force divided by the box area, unit: Pa) and the displacement field $\mathbf{u}(x, y, t)$ is

$$\boldsymbol{u} = \boldsymbol{G} * \boldsymbol{T}, \tag{S35}$$

where $\boldsymbol{G}$ is the Green's function and $*$ represents convolution in space. In practice, the calculations are performed in Fourier space, so we actually calculated

$$\tilde{\boldsymbol{T}} = \left(\tilde{\boldsymbol{G}}^T\tilde{\boldsymbol{G}} + \lambda^2\boldsymbol{I}\right)^{-1}\tilde{\boldsymbol{G}}^T\tilde{\boldsymbol{u}}, \tag{S36}$$

where $\tilde{\boldsymbol{T}}$ and $\tilde{\boldsymbol{u}}$ are the Fourier transform of $\boldsymbol{T}$ and $\boldsymbol{u}$, respectively, $\tilde{\mathbf{G}}$ is the Fourier-transformed Green's function

$$\tilde{G}_{ij}(k_x, k_y) = \frac{2(1+\nu)}{E(k_x^2 + k_y^2)^{3/2}}\begin{pmatrix}(k_x^2 + k_y^2) - \nu k_x^2 & -\nu k_x k_y \\ -\nu k_x k_y & (k_x^2 + k_y^2) - \nu k_y^2\end{pmatrix}, \tag{S37}$$

$\boldsymbol{I}$ is the unit matrix, the Poisson's ratio $\nu \approx 0.5$, and λ is the regularization parameter. In the end, the inverse Fourier transformation of $\tilde{\boldsymbol{T}}$ gives us the traction map $\boldsymbol{T}$.

D. Optimizing the regularization parameter

The optimized regularization parameter λ can be estimated using the ratio between the standard deviation of displacement σ_u when there is no external force applied and the characteristic traction scale of interest σ_T

[15]. As λ increases, the noise will be attenuated more significantly, but on the other hand, the magnitude of the actual signal is suppressed. This trade-off motivates us to take a closer look at how λ affects the final result, so we generated some artificial traction maps (Fig. S23a), calculated the corresponding displacement field using Eq. S35, added Gaussian noise with standard deviation σ_u to the displacement (Fig. S23b), and reconstructed the traction using our TFM analysis algorithm (Fig. S23c). The parameters we chose in the calculation matched those in the experiments.

We measured the noise level of displacement using bare gels without any bacterium on the surface. The distribution of u_x and u_y on such bare gel surfaces are shown in Fig S23d for 60× magnification, and they each had a standard deviation of $\sigma_u \approx 4.5$ nm. The traction $\boldsymbol{T}$ generated by $\Delta pilA$ mono-layers is discussed in detail in the main text. The distribution of its magnitude $|\boldsymbol{T}| \equiv T$ has a characteristic width $\sigma_T \approx 10$ Pa, so our expected regularization parameter is $\lambda = \sigma_u/\sigma_T \approx 0.5$ nm/Pa. In the calculations shown in Fig. S23a-c, the input traction field had eight circular regions where forces parallel to the surface were applied. Each region had a radius of $r_f = 2.5$ μm with constant traction of 30 Pa applied uniformly, and then we smoothed their edges with a moving Gaussian filter (the standard deviation of this Gaussian window was $r_f/4$). We varied the peak value of the input traction $T_{\text{in, max}}$ and calculated the ratio T/T_{in} in the regions where $T_{\text{in}} > 0.5T_{\text{in, max}}$ with different λ, as shown in Fig. S23e. We used two parameters to evaluate the effect of λ: the input traction at which $T/T_{\text{in}} = 1$ and the average ratio in the flat region on the right-hand side $\overline{(T/T_{\text{in}})}_{\text{flat}}$. An ideal λ should reduce the noise as much as possible while keeping T/T_{in} close to 1. As shown in Fig. S23f, $\lambda \in [0.4, 1]$ nm/Pa is the preferred range, and we chose $\lambda = 0.6$ nm/Pa as the regularization parameter when analyzing the experimental data.

VII. DEFORMATION NORMAL TO THE SUBSTRATE SURFACE

A. Measuring normal deformation of the substrate surface

Looking at the fluorescent images, we noticed that the brightness of the particles decreased when a thicker layer of cells moved across that area. As shown in Fig. S24a and b, a double layer of cells moved across the field of view from the lower left corner to the upper right corner, and it caused a shadow in the fluorescent images that moved with it. We used this variation in brightness to measure the deformation of the substrate surface in the normal (z) direction (Fig. S24c). The fluorescent brightness is controlled by the surface deformation because when imaging the time series, our focal plane was right at the surface of the substrate, as illustrated in Fig. S24e. As the surface deformed downward, less light emitted by the fluorescent particles was captured at the focal plane, so this region became darker. Similarly, when the surface deformed upward, the light emitted by the particles on both sides of the focal plane was captured, so this region became brighter. We divided the fluorescent images with a square lattice with a 20×20 pixel^2 box size and used the average brightness within each box to represent the local brightness. The average brightness in two of such boxes (labeled in Fig. S24b) is shown in Fig. S24d, where the colors of the curves and the boxes match. The figure shows that the double-layered region both reached and left the cyan box earlier than the magenta box.

To convert this brightness variation into z displacement, when imaging, we took a z-stack at the same location immediately after taking each time series. In the time series, we took consecutive images at the same z (15 s between adjacent frames), while in the z-stack, we imaged different slices in z at a rate of about 5 s per slice. Most z-stack images were taken from tens of microns below the substrate surface to several microns above, with a step size of $\Delta z = 0.2$ μm. We also took z-stacks that covered the whole substrate thickness with 2 μm step size. In both the time series and z-stack, we took a bright field image and one or more fluorescent images (one for each necessary fluorescent color) at each time or z step. From the z-stack, we calculated the average brightness as a function of z (Fig. S24f) with multiple videos of $\Delta pilA$ cells, in which the cells formed a monolayer with occasional double-layers or holes. The brightness decreases as z increases, and its slope steepens near the substrate surface. For each experiment, we linearly fitted the section with the steepest slope and the section above the substrate surface (the section on the right), as shown by the black dashed lines in Fig. S24f. Their intersection (z_0, I_0) was used to align the curves in the horizontal direction ($z \to z - z_0$) and normalize the brightness ($I \to I/I_0$). The normalized $I(z)$ curves align well with each other, and Fig. S24f shows their mean and standard deviation. The imaging plane in the time series is indicated by the vertical black line. Above this plane (up in z), the $I(z)$ curve remains approximately linear across a range of about 1.5 μm. The red line is a linear fitting of the data in this regime, and its slope k_I maps the brightness variation in the time series to surface deformation:

$$\Delta z = \Delta I/k_I. \tag{S38}$$

The resulting measurement of z in the time series is shown in Fig. S24c, where the z position below a monolayer of cells was defined as $z = 0$ μm. A double layer of cells led to negative z, and a hole in the cell layer led

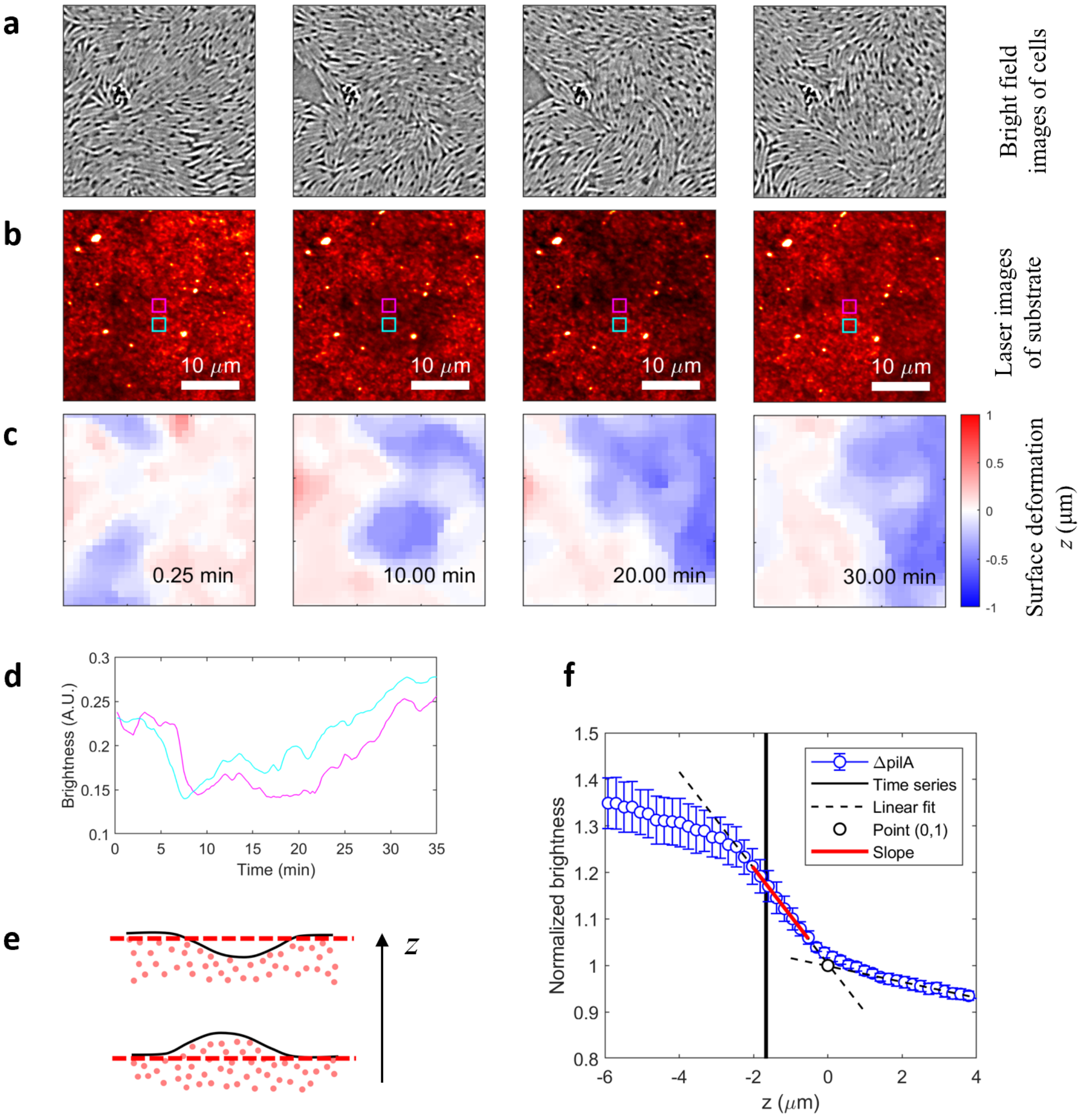

Fig. S24: Measuring substrate deformation perpendicular to the surface (z). (a-c) Bright-field images of the cells (a), fluorescence images of the fluorescent beads (40 nm) in the substrate (b), and measured surface deformation in z at four different times. Images in the same column correspond to the same time as those labeled in (c). The scale bars in (b) indicate 10 μm. (d) The average brightness as functions of time in the two boxes labeled in (b). (e) Sketches of the relative positions of the fluorescent particles (red dots) and the focal plane (red dashed lines) when the surface of the substrate (black curves) deforms down or up. (f) Normalized brightness of z-stack images of the fluorescent particles as a function of z. The blue data were from 12 experiments with $\Delta pilA$ cells on the substrate. The dashed black lines are the linear fits of the two approximately linear regions near and above the substrate surface. Using the slope of the thick red line, we converted the variation in brightness to the difference in z in the time series, where only one slice in z was imaged. The solid black line shows the average position of this slice in the time series.

to positive z. As discussed in the main text, this is because the surface tension at the gel-cell-air interface is stronger than the stiffness of the substrate, so the interface remained relatively flat, and a "second" layer of cells grew below the "first" layer.

B. Zero traction using layer information

To calculate the traction applied on the substrate, we need to know the location of the particles in the substrate when there is no force applied. However, since our system is not driven by cell growth and we could not predict the cell motion, it was difficult for us to start by imaging an area without any cells and wait for the cells to migrate over and form a monolayer. Instead, we always imaged regions with cells already in frame one and thus did not have the "zero displacement" state of the substrate. The consequence is that for each pixel in the reconstructed traction map, there is an unknown constant that represents the traction applied at that location in the first frame. As shown in Fig. S25a, the exemplary particle is at position A (dashed green circle)

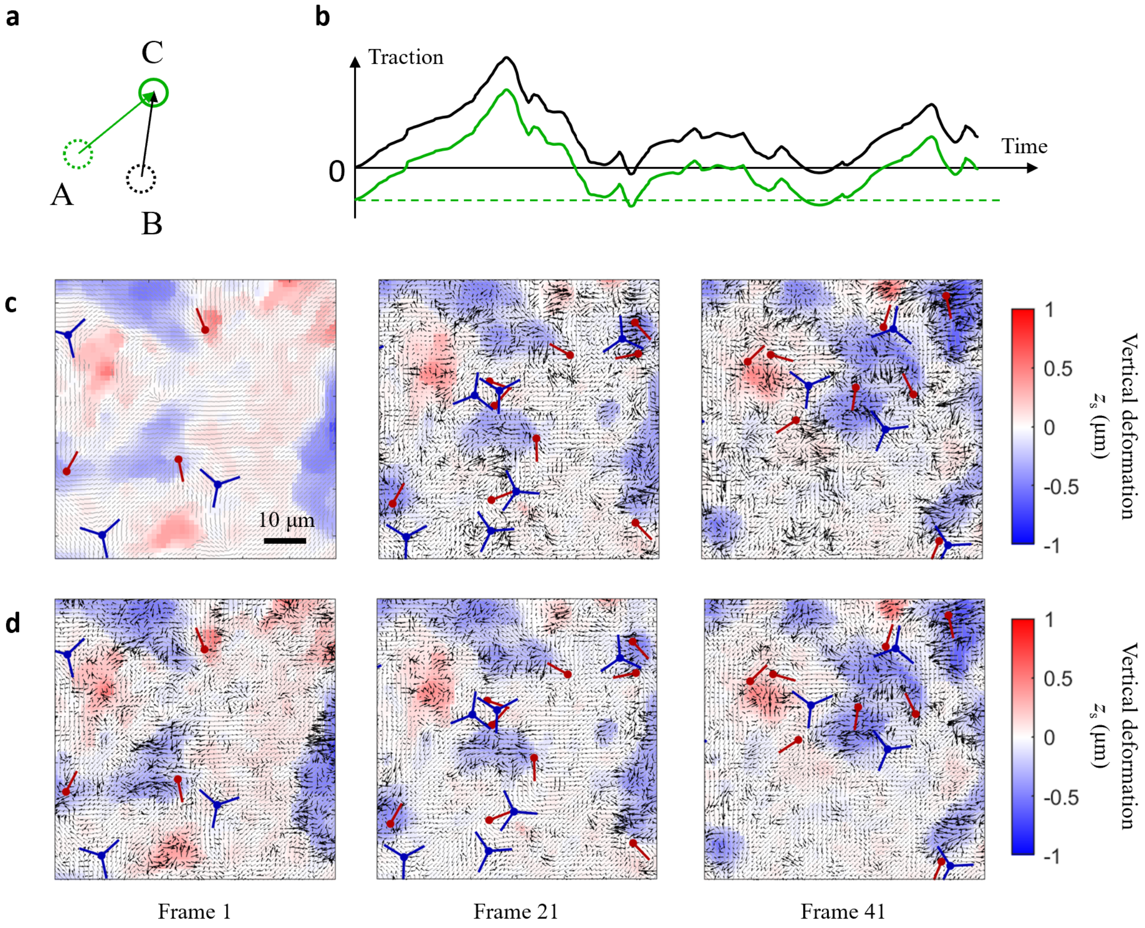

Fig. S25: Separating the DC mode from raw traction. (a) A sketch of the problem: when there is no force applied, the particle in the substrate is at point A (dashed green circle). In Frame 1, it is at point B because the substrate is not traction free. In a later frame, the particle moves to point C. The green arrow shows its displacement with respect to A, and the black arrow is with respect to B. At each location, given (b) A sketch of the measured traction (black line) and the actual traction (green line) as functions of time. The dashed green line labels the DC component that is removed. (c) Raw traction using frame 1 as the reference. The color map shows z deformation of the substrate surface. The scale bar represents 10 μm. (d) Traction with the DC component removed.

when no force applies on the substrate. However, in the first frame of the video, since there is some non-zero traction applied at this location, it has moved to position B (dashed black circle). When we calculate its future displacement using frame one as the reference frame, for example, when the particle moves to position C (solid green circle), we obtain the black arrow instead of the green arrow (correct displacement). Consequently, at each location, there is an unknown 0 Hz frequency mode (DC component) in the measured time series of traction (Fig. S25b), and the value of this constant varies pixel by pixel.

One way to remove this unknown DC mode is by setting a threshold frequency and separating the high-frequency traction from the low-frequency component. The DC mode is part of the low-frequency component and thus is removed. However, according to Fig. 4e of the main text, there is no characteristic frequency that justifies the value of this frequency threshold. Moreover, removing the low-frequency component flattens the traction jump when a monolayer to double-layer transition happens as shown in Fig. 3 of the main text.

In this paper, we separated the DC component from the total traction $\boldsymbol{T}$ by taking the normal deformation z into consideration as well. When the number of cell layers remained constant, the traction fluctuated around a mean value. So at pixel (i, j), we found the frames in which $z_{ij} \in [-0.1\ 0.1]$ μm, calculated the mean traction $\langle \boldsymbol{T}_{ij} \rangle$ in these frames, and removed it from the total traction $\boldsymbol{T}_{ij}$ pixel by pixel. This way we removed the DC component and kept the traction jump when double layers formed. Fig. S25c shows the raw traction $\boldsymbol{T}_{ij}$ using frame 1 as the reference frame. Fig. S25d shows the traction with the DC components removed $\boldsymbol{T}_{ij} - \langle \boldsymbol{T}_{ij}(-0.1 \leq z_{ij} \leq 0.1) \rangle$. Note that when a hole became a monolayer, or vice versa, there was a traction variation as well. Ideally, the traction in a hole is zero, and it should increase when cells migrate into this hole. However, when using the traction below monolayers as the reference, we got the opposite, which can be seen in Fig. S25d: in the hole near the bottom edge of frame 1 (red region), the traction was non-zero. When the hole was filled later by cells, the local traction nearly vanished. Nevertheless, since in this paper, we focus on the

monolayer to double-layer transitions, this is not a problem.

VIII. SUPPLEMENTARY TABLES

Shear modulus (Pa)	PAA Stock (ml)	Water (ml)	Bead suspension (μl)	10% APS (μl)	Temed (μl)
230	0.03	0.2185	10	1.25	0.375
430	0.05	0.1985	10	1.25	0.375
1500	0.075	0.1735	10	1.25	0.375

TABLE S3: Formulae for making 0.25 ml PAA hydrogels using the PAA stocks. APS stands for ammonium persulfate solution in water.

Shear modulus (Pa)	40% Acrylamide (ml)	2% Bis (ml)	Water (ml)
230	3.13	1.25	0.63
430	3.13	0.63	1.25
1500	3.13	0.42	1.46

TABLE S4: Formula for making 5 ml PAA stocks.

IX. SUPPLEMENTAL MOVIES

Supplemental Movie 1
Data obtained from the traction force microscopy (TFM) experiments. We used bright field images of the cells (left panel) to measure the director field (orange bars) and cell velocity field (not shown in this video). With the director field, we obtained the locations and orientations of $+1/2$ (red labels) and $-1/2$ (blue labels) defects in the cell layer. We used laser images of the fluorescent particles in the substrate (middle panel) to measure the tangential surface deformation of the substrate. The white arrows show the displacement field, based on which we reconstructed the traction force field applied on the substrate surface by the cells (right panel). The color map represents the magnitude of the traction field, and the black arrows show the directions and magnitudes of the local traction.

Supplemental Movie 2
Polarity measurement with a single cell. The left panel is in the lab frame and the right panel is in the co-moving frame of the cell. The MglB protein in the cell is labeled in red and the pink arrow shows the velocity of the cell.

Supplemental Movie 3
Polarity measurement in a cell monolayer. The bright field images show the cells and the red dots label the MglB proteins.

Supplemental Movie 4
Using particle brightness to measure surface deformation, which is produced by the formation of cell layers. The left panel shows the bright field images of the cells. The middle panel shows the corresponding laser images of the fluorescent particles embedded in the substrate in the same area. The right panel shows the surface deformation in the z direction (perpendicular to the undeformed surface) calculated using the brightness of the particles. When a second layer of cells formed, the substrate surface was pushed down, and the brightness of the particles decreased in that region.

Supplemental Movie 5
Second layer formation near a $+1/2$ defect. The white circle has a radius of 12 μm. On the right-hand side, we show the total topological charge inside the white circle and the change in volume ΔV. The open circles in these two plots label the time corresponding to the image on the left. The time $t = 0$ min is defined as the time when a visible second layer appeared in the bright field images.

Supplemental Movie 6
Following a $+1/2$ defect not leading to second layer formation. The plots and labels are identical to Movie 5. Here the time $t = 0$ min was chosen randomly during the period when the topological charge inside the circular area was $+1/2$.

Supplemental Movie 7
Traction variation when a second layer forms near a $+1/2$ defect. The left panel shows the bright field images of the cells and the right panel shows the number of cell layers in the same region with the color map. The yellow bars label the director field and the black arrows show traction.

[1] K. Copenhagen, R. Alert, N. S. Wingreen, and J. W. Shaevitz. Topological defects promote layer formation in myxococcus xanthus colonies. *Nature Physics*, 17(2):211–215, 2021.

[2] M. E. Black and J. W. Shaevitz. Rheological dynamics of active myxococcus xanthus populations during development, 2021.

[3] J. Zhu, L. Chen, J. Shen, and V. Tikare. Coarsening kinetics from a variable-mobility cahn-hilliard equation: Application of a semi-implicit fourier spectral method. *Physical Review E*, 60(4):3564, 1999.

[4] B. Qin, C. Fei, A. A. Bridges, A. A. Mashruwala, H. A. Stone, N. S. Wingreen, and B. L. Bassler. Cell position fates and collective fountain flow in bacterial biofilms revealed by light-sheet microscopy. *Science*, 369(6499):71–77, 2020.

[5] Daniel Wall and Dale Kaiser. Type iv pili and cell motility. *Molecular Microbiology*, 32(1):01–10, 1999.

[6] E. M. Mauriello, T. Mignot, Z. Yang, and D. R. Zusman. Gliding motility revisited: how do the myxobacteria move without flagella? *Microbiol Mol Biol Rev*, 74(2):229–49, 2010.

[7] B. Sabass, M. D. Koch, G. Liu, H. A. Stone, and J. W. Shaevitz. Force generation by groups of migrating bacteria. *Proc Natl Acad Sci U S A*, 114(28):7266–7271, 2017.

[8] C. Kaimer and D. R. Zusman. Regulation of cell reversal frequency in myxococcus xanthus requires the balanced activity of chey-like domains in frze and frzz. *Mol Microbiol*, 100(2):379–95, 2016.

[9] D. Szadkowski, L. A. M. Carreira, and Lotte Søgaard-Andersen. A bipartite, low-affinity roadblock domain-containing gap complex regulates bacterial front-rear polarity. *bioRxiv*, page 2022.03.17.484758, 2022.

[10] H. Li, X. Q. Shi, M. Huang, X. Chen, M. Xiao, C. Liu, H. Chate, and H. P. Zhang. Data-driven quantitative modeling of bacterial active nematics. *Proc Natl Acad Sci U S A*, 116(3):777–785, 2019.

[11] A. J. Vromans and L. Giomi. Orientational properties of nematic disclinations. *Soft Matter*, 12(30):6490–5, 2016.

[12] D. Blair and Dufresne E. The matlab particle tracking code repository. *Particle-tracking code available at site.physics.georgetown.edu/matlab/*, 2018.

[13] N. G. Deen, P. Willems, M. van Sint Annaland, J. A. M. Kuipers, R. G. H. Lammertink, A. J. B. Kemperman, M. Wessling, and W. G. J. van der Meer. On image pre-processing for piv of single- and two-phase flows over reflecting objects. *Experiments in Fluids*, 49(2):525–530, 2010.

[14] B. Sabass, M. L. Gardel, C. M. Waterman, and U. S. Schwarz. High resolution traction force microscopy based on experimental and computational advances. *Biophys J*, 94(1):207–20, 2008.

[15] S. V. Plotnikov, B. Sabass, U. S. Schwarz, and C. M. Waterman. High-resolution traction force microscopy. *Methods Cell Biol*, 123:367–94, 2014.

[16] U. S. Schwarz and J. R. Soine. Traction force microscopy on soft elastic substrates: A guide to recent computational advances. *Biochim Biophys Acta*, 1853(11 Pt B):3095–104, 2015.

[17] L.D. Landau and E.M. Lifshitz. *Theory of Elasticity (Second Edition)*. Pergamon Press, 1970.
