# Supplementary material for: Local polar order controls mechanical stress and triggers layer formation in Myxococcus xanthus colonies
Source: Nat Commun. 2025 Jan 22;16:952. doi: 10.1038/s41467-024-55806-6 (PMC11754464; doi:10.1038/s41467-024-55806-6)
Supplement: Supplementary file 2 — Description of Additional Supplementary Files [file 41467_2024_55806_MOESM2_ESM.pdf]

Supplemental Movie 1
Data obtained from the traction force microscopy (TFM) experiments. We used bright field images of the cells (left panel) to measure the director field (orange bars) and cell velocity field (not shown in this video). With the director field, we obtained the locations and orientations of +1/2 (red labels) and -1/2 (blue labels) defects in the cell layer. We used laser images of the fluorescent particles in the substrate (middle panel) to measure the tangential surface deformation of the substrate. The white arrows show the displacement field, based on which we reconstructed the traction force field applied on the substrate surface by the cells (right panel). The color map represents the magnitude of the traction field, and the black arrows show the directions and magnitudes of the local traction.

Supplemental Movie 2
Polarity measurement with a single cell. The left panel is in the lab frame and the right panel is in the co-moving frame of the cell. The MglB protein in the cell is labeled in red and the pink arrow shows the velocity of the cell.

Supplemental Movie 3
Polarity measurement in a cell monolayer. The bright field images show the cells and the red dots label the MglB proteins.

Supplemental Movie 4
Using particle brightness to measure surface deformation, which is produced by the formation of cell layers. The left panel shows the bright field images of the cells. The middle panel shows the corresponding laser images of the fluorescent particles embedded in the substrate in the same area. The right panel shows the surface deformation in the z direction (perpendicular to the undeformed surface) calculated using the brightness of the particles. When a second layer of cells formed, the substrate surface was pushed down, and the brightness of the particles decreased in that region.

Supplemental Movie 5
Second layer formation near a +1/2 defect. The white circle has a radius of 12 μm. On the righthand side, we show the total topological charge inside the white circle and the change in volume ΔV. The open circles in these two plots label the time corresponding to the image on the left. The time $t = 0$ min is defined as the time when a visible second layer appeared in the bright field images.

Supplemental Movie 6
Following a +1/2 defect not leading to second layer formation. The plots and labels are identical to Movie 5. Here the time $t = 0$ min was chosen randomly during the period when the topological charge inside the circular area was +1/2.

Supplemental Movie 7
Traction variation when a second layer forms near a +1/2 defect. The left panel shows

the bright field images of the cells and the right panel shows the number of cell layers in the same region with the color map. The yellow bars label the director field and the black arrows show traction.
